# Nucleo-amino acid derived AMO as a potential JAK inhibitor: machine learning screening, docking, molecular dynamics, ADMET/toxicity, and MM-PBSA analysis

**DOI:** 10.1016/j.crstbi.2026.100191

**Published:** 2026-06-15

**Authors:** Morteza Hosseini, Alireza Fattahi

**Affiliations:** Department of Chemistry, Institute for Convergence Science and Technology, Bioinformatics Group, Sharif University of Technology, Tehran, Iran

**Keywords:** L-amino acids, Nucleotide bases, Abrocitinib, Machine learning, Molecular docking, ADMET prediction, Pre-tox analysis, Molecular dynamics simulation, MM-PBSA analysis

## Abstract

L-amino acids and nucleotide bases are essential biomolecules in all living organisms and represent attractive building blocks for designing safer, more effective drugs. In this study, we designed JAK kinase inhibitor candidates derived from these natural compounds and evaluated them using an integrated computational workflow. MarvinSketch was used to generate 100 structures, which were optimized in Spartan; frequency calculations confirmed the absence of imaginary modes and enabled calculation of key physicochemical properties. The candidates were then screened against five known JAK inhibitors using machine learning models (SVR, RF, KNN, and an ensemble approach), yielding (3S,5S)-5-((2-amino-6-oxo-1,6-dihydro-9H-purin-9-yl)methyl)-3-(4-aminobutyl)morpholin-2-one (AMO) as the top hit. Molecular docking identified favorable active-site interactions, and Abrocitinib (ABR) was selected as the closest comparator with the best overlay to AMO. Molecular dynamics simulations showed stable AMO binding, supported by RMSD/RMSF, radius of gyration, hydrogen-bonding, and DSSP analyses. PCA indicated a more compact conformational distribution for AMO than ABR, consistent with reduced large-scale motions, and DCCM supported favorable correlated dynamics. Pharmacokinetic profiling and ProTox-3.0 toxicity predictions classified both compounds as class 4, but AMO exhibited substantially lower predicted toxicity than ABR. Finally, MM-PBSA calculations yielded negative binding energies, confirming that the AMO–protein interaction is thermodynamically favorable and strong.

## Highlights

• Nature-inspired design strategy: created a new scaffold by combining L-amino acids & nucleotide bases to pursue higher efficacy and fewer side effects in JAK inhibition.
• A comprehensive computational framework that integrates quantum optimization, machine learning screening, and molecular docking has identified AMO as the leading candidate.
• MD analyses (RMSD, RMSF, Rg, H-bonds, DSSP) confirmed stable AMO binding in the JAK active site, while PCA and DCCM showed a more compact and favorable conformation than ABR.
• ADMET and MM-PBSA analyses indicated lower toxicity and strong binding for AMO.

## Graphical abstract

**Figure:**
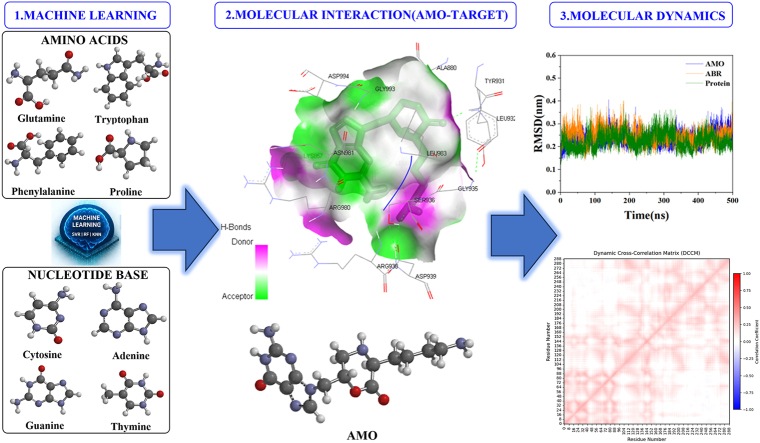

## 1 Introduction

**Fig. 1:**
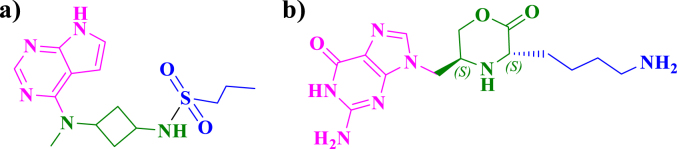
The chemical structure of (a) **ABR** and (b) **AMO**.

L-amino acids and nucleotide bases are highly valuable biomolecules in living organisms. As the building blocks of DNA and RNA, nucleotides preserve and transmit genetic information. Furthermore, in addition to their essential role in protein synthesis and cellular processes, amino acids are highly significant in the development and efficacy of drugs; recently, they have been utilized in the development of novel immunogenic adjuvants (such as squalene-based polyamino acids) (Lim et al., 2019; Zhang et al., 2023). Furthermore, Co-administering this combination with the inactivated H1N1 vaccine boosted immune responses (antibodies and T-cells) in mice, providing complete protection against disease symptoms and fatal infection (Wang et al., 2016). L-amino acids have been used to produce antimicrobial peptides (Pfalzgraff et al., 2018) and to enhance drug efficacy; for instance, forming Trimethoprim salts with aspartic or glutamic acid significantly improves its absorption and solubility (ElShaer et al., 2012; Gao et al., 2019). Due to their high-water solubility and broad activity, amino acids are used to modify and enhance poorly soluble bioactive compounds (Xu et al., 2021). In addition, amino acids play main roles as intermediates and catalysts in organic chemistry (List et al., 2000). Also, L-amino acids are widely used as chiral inducers and organic catalysts in asymmetric and multicomponent synthesis reactions due to their chirality (Thorat et al., 2023; Karmakar and Mukhopadhyay, 2024). Moreover, L-amino acids are of high importance in drug design and development. Recently, the use of L-amino acid-based compounds in drug design and discovery has received attention. In a study, Kalhor et al. (Kalhor and Fattahi, 2022). reported the design of an amino acid-based anticancer drug targeting the polymerase η enzyme. Janus kinases (JAK1, JAK2, JAK3, and TYK2) are crucial enzymes that regulate immune and inflammatory responses through the JAK-STAT signaling pathway. JAK inhibitors target this pathway by blocking cytokine signals, effectively reducing immune system overactivity, inflammation, and pain associated with various diseases (Clark et al., 2014; Yao et al., 2018). The JAK-STAT pathway is crucial for immune system development, blood cell formation, and tissue homeostasis, but its abnormal activation can lead to autoimmune disorders and cancers. Consequently, small-molecule JAK inhibitors have become highly effective treatments for conditions like rheumatoid arthritis, inflammatory bowel disease, and myeloproliferative neoplasms. Current research focuses on developing next-generation, highly selective JAK inhibitors to maximize therapeutic benefits while minimizing off-target side effects (Banerjee et al., 2017). Therefore, in this study, novel structures derived from L-amino acids and nucleotide bases were designed and evaluated as potential JAK kinase inhibitors. The computational workflow involved predicting molecular structures using MarvinSketch, optimizing energy and extracting physical properties via Spartan, and finally screening 100 proposed structures alongside 5 reference drugs using various machine learning models (SVR, RF, KNN, and Ensemble). Afterward, Molecular docking analysis using AutoDock Tools identified the AMO structure as the best candidate due to its optimal binding interactions and high compatibility with ABR. This is a highly selective, oral JAK1 inhibitor that mitigates inflammation and itchiness by blocking the JAK-STAT signaling pathway. However, because it suppresses the immune system, ABR carries mild side effects like headaches and nausea, alongside severe risks such as infections, blood clots, and cardiovascular diseases (Gooderham et al., 2021). Molecular dynamics (MD) simulations, ADMET predictions, pre-Tox analysis, and MM-PBSA calculations were performed for the drug and AMO structures. Fig. 1 shows the structures of (a) ABR and (b) AMO. Based on the computational results, this alternative candidate is expected to be safer and have fewer side effects compared to ABR due to the use of natural starting materials in its structure, and to act as a potential JAK enzyme inhibitor. Further experimental evaluations and clinical trials are required to confirm its efficacy and therapeutic potential.

## 2 Method and theoretical calculations

### 2.1 The structural model

**Fig. 2:**
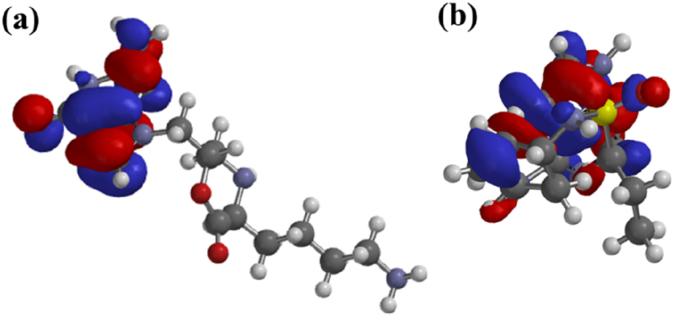
Optimized geometries and corresponding highest occupied molecular orbital (HOMO) energies. (a) AMO and (b) ABR at the EDF2/6-31G(d) level.

Since only twenty L-amino acids constitute the proteins of the human body, together with the five nucleotide bases that participate in the structure of DNA and RNA, a total of one hundred possible structures were estimated using MarvinSketch (Ghersi and Singh, 2014). In addition to generating high-resolution chemical drawings, this software can also predict the number of possible structures based on the number of components in the initial configuration. Each structure can adopt multiple molecular conformers. Initial conformational searches were performed using the MMFF (Molecular Mechanics Force Field) in Spartan software (Shao et al., 2011; Chanda and Harohally, 2018) to obtain the most stable conformer for subsequent energy optimization. Single-point energy calculations, conducted to ensure the absence of imaginary frequencies, were performed on the optimized structures at the EDF2/6-31G(d) level (in vacuum) using Spartan. This method is designed for the accurate prediction of vibrational frequencies; unlike other approaches such as B3LYP, it typically does not require the application of scaling factors and provides highly reliable thermochemical data. Moreover, it allows for faster computation. These calculations were used to obtain physical properties such as volume, surface area, polar surface area, and polarizability for the five drugs and the designed structures. In the next step, five JAK kinase inhibitor drugs and one hundred proposed structures were screened using machine learning models (SVR, RF, KNN, and Ensemble). The structure AMO was selected as the most promising candidate. Computational approaches were employed to investigate the binding potential of the selected compounds with the target protein. Prior to docking analysis, the protein structure was prepared and optimized to ensure accurate modeling of the binding environment. In addition, ligand structures were energy-minimized to obtain stable conformations suitable for docking studies. Subsequently, molecular docking was performed using AutoDock to identify the most effective interaction in the protein's active site, and ABR was identified as the best drug candidate. Fig. 2 presents the highest occupied molecular orbital (HOMO) geometries of the AMO and ABR conformers, while Table 1 and S1summarize the physical properties and binding energies of the proposed structures and the five JAK kinase inhibitor drugs.

**Table 1:** Information and physical properties of JAK enzyme inhibitor drugs and the proposed designed structure based on amino acid and nucleotide base (AMO) whose affinity has been calculated based on the ABR.

Entry	Name and PDB ID	Drug	Volume(Å^3^)	Area (Å^2^)	Polarizability	PSA^a^(Å^2^)	BE^b^ (Kcal/mol)
1	Delgocitinib-7C3N		311.41	330.53	64.03	58.62	−8.9
2	Baricitinib-6WTO		345.85	375.98	66.96	90.99	−8.1
3	Tofacitinib-3EYG		319.10	336.30	64.66	57.31	−8.9
4	Decernotinib-4YTI		364.89	389.93	68.54	58.46	−10.0
5	Abrocitinib (ABR)-6BBV		315.88	351.01	64.40	75.33	−8.1
6	AMO		314.82	332.29	64.24	115.68	−8.4

a Polar Surface Area.

b Binding energy.

For further investigation, molecular dynamics (MD) simulations and MM-PBSA calculations, finally, ADMAT predictions, Pre-Tox (Banerjee et al., 2018) analysis (https://tox.charite.de/protox3/), were performed for the ABR and AMO structures and compared.

### 2.2 Machine learning

A machine learning approach was employed to investigate the similarity between the predicted molecular structures and reference JAK inhibitor drugs. The dataset consisted of 100 predicted molecular structures described by physicochemical descriptors obtained from quantum chemical calculations. Structural similarity between the predicted compounds and the reference drugs was estimated using Euclidean distance calculated from the corresponding molecular descriptors, and the resulting values were used as similarity indices for modeling (Cherkasov et al., 2014). Prior to model development, all descriptors were standardized to eliminate scale differences among variables (Tropsha, 2010). Several machine learning algorithms commonly applied in cheminformatics and drug discovery studies were used for modeling, including Support Vector Regression (SVR)(Cortes and Vapnik, 1995), K-Nearest Neighbors (KNN), and Random Forest (RF). These algorithms were selected because they represent different modeling strategies for capturing relationships within molecular descriptor datasets. SVR applies kernel-based functions to model nonlinear relationships between variables, KNN is a similarity-based non-parametric method that predicts values according to neighboring samples in feature space, and RF is an ensemble learning method that constructs multiple decision trees to improve prediction robustness and reduce overfitting (Breiman, 2001). In addition, an ensemble approach was used to integrate the predictions obtained from the individual models in order to improve model stability. Model performance was assessed using standard statistical metrics including Mean Squared Error (MSE), Mean Absolute Error (MAE), Root Mean Squared Error (RMSE), and the coefficient of determination (R^2^). To ensure the robustness of the machine learning models and mitigate the risk of overfitting particularly given the dataset size of 100 molecules K-fold cross-validation technique (K = 5) was strictly employed during the model training and evaluation phase. The dataset was randomly partitioned into 5 equal-sized subsamples. In each iteration, one subsample was retained as the validation set for testing the model, while the remaining 4 subsamples were used as training data. This process was repeated 5 times, with each of the subsamples used exactly once as the validation data. The final performance metrics (e.g., R^2^, MSE) were calculated as the average of the results from all folds, ensuring that the model's predictive capability is generalized and not dependent on a specific random data split. All analyses were performed in Python using commonly applied scientific libraries (Pedregosa et al., 2011).

### 2.3 Molecular docking

Molecular docking studies were performed using AutoDock Vina (Gaillard, 2018) to evaluate binding energies between a variety of drugs and their respective proteins, using PDB codes (http://www.pdb.org). For each drug, a grid box was defined to encompass the binding site: Delgocitinib (Noji et al., 2020) (PDB ID: 7C3N) with dimensions 22 × 20 × 40 Å centered at (8.63, −2.417, 15.028); Abrocitinib (Vazquez et al., 2018) (PDB ID: 6BBV) with dimensions 26 × 20 × 26 Å centered at (−0.167, 1.83, 4.89); Baricitinib (Chang et al., 2021) (PDB ID: 6WTO) with dimensions 36 × 26 × 24 Å centered at (−9.6, −7.20, 5.52); Tofacitinib (Williams et al., 2009) (PDB ID: 3EYG) with dimensions 22 × 26 × 28 Å centered at (9.6, 6.2, −5.7); and Decernotinib (Farmer et al., 2015) (PDB ID: 4YTI) with dimensions 22 × 28 × 20 Å centered at (8.06, 6.52, 1.08). The protein structure of each drug was prepared using AutoDock Tools (Huey et al., 2012), incorporating polar hydrogens and assigning fractional charges using the Coulombic method, and then saved in the pdbqt format. Ligands were similarly processed by adding polar hydrogens and calculating partial charges, and then saved in the pdbqt format. This was done for all five drugs and compared with the AMO structure that had been screened and selected from the previous step. Then, considering the binding energy and to achieve the most effective interactions in the active site of the protein, finally, ABR (PDB ID: 6BBV) and AMO showed the highest molecular compatibility and overlay and were selected for further analysis.

### 2.4 ADMET prediction & Pre-Tox analysis

To predict the physicochemical and pharmacokinetic properties of both ABR and AMO compounds, the SwissADME (Daina et al., 2017) online web server (https://www.swissadme.ch/) was used to evaluate and compare them. According to Lipinski's rule of five (Benet et al., 2016)^,^ which examines the solubility and permeability properties of compounds and predicts their bioavailability as drug candidates. Accordingly, compounds that violate Lipinski's rule of five are likely to have inadequate absorption or penetration. Therefore, this study used in silico computational approaches, which is a common approach to understand and predict these properties in the early stages of drug design and discovery. To accurately predict pharmacokinetic interactions, the toxicity effects of both compounds were investigated using the Pro-Tox 3.0 web server. Since this method, in addition to assessing molecular similarity, also examines more than 45 endpoints, including acute toxicity, it can cover a more detailed assessment of metabolism and examine precise descriptors.

### 2.5 Molecular dynamics simulation

The molecular dynamics (MD) simulation of the studied complex was performed using the GROMACS 2025.3 software package (https://www.gromacs.org/)(Jangid et al., 2025). For the water solvent model (TIP3P), the simulation boxes were defined as cubes with all faces separated by 1.0 nm. The force field is also CHARMM 36 (Yekeen et al., 2023). Ligand parameterization was performed using the official CHARMM General Force Field server and using the CGenFF tool (https://cgenff.com/). Initial energy minimization was performed using the steepest descent algorithm to avoid steric collisions and lower the system's potential energy. After energy minimization, a two-step equilibration process was performed. Then, focal group-assisted (NVT) and isothermal-isobaric (NPT) equilibration steps were performed using the Leap-frog algorithm (Jiang et al., 2025) for 500,000 integration steps. The Noz-Hoover thermostat constant was used to keep the system temperature constant at 310 K. The Parinello-Rahman pressure coupling method (Saito et al., 2003) was used to keep the system pressure constant at 1 bar. Finally, molecular dynamics simulations were run in extended simulation mode for 500 ns. The results of these simulations show that AMO exhibits a much better dynamic stability profile compared to ABR. These simulations focus specifically on ligand binding to a target protein.

### 2.6 MM-PBSA analysis to estimate the binding affinity

The molecular mechanics Poisson-Boltzmann surface area (MM-PBSA) method (Mandal and Mandal, 2025) is widely used to estimate free energy changes in protein-ligand interactions. In this method, ΔG_binding_ is given by the relationship ΔG_binding_ = G_complex_ – (G_protein_ + G_ligand_), where ΔG_binding_ is the binding free energy. The solvation free energy (ΔG_solv_) is estimated as the sum of the polar contribution to the solvation free energy (ΔG_PB_) calculated using the implicit Poisson-Boltzmann solvent model, and the nonpolar contribution is calculated based on the solvent accessible surface area (ΔG_SA_). The implicit Poisson-Boltzmann solvent model estimates the polar solvation free energy by treating the solvent as a continuous, uniform medium without explicit solvent molecules and accounting for interactions between the solute atoms and the implicit solvent. This analysis uses the MM-PBSA framework to decompose the binding free energy into molecular mechanics, solvent-accessible surface area, and nonpolar contributions. Free energies were calculated using the gmxMMPBSA module at 310 K in the last frames, 50,000 times, for a total of 500,000,000 to perform this analysis. The output data, expressed in DAT files, are processed using Python scripts to determine the specific residual energy contribution and calculate the net binding free energy.

## 3 Results and discussion

### 3.1 Machine learning based screening

**Fig. 3:**
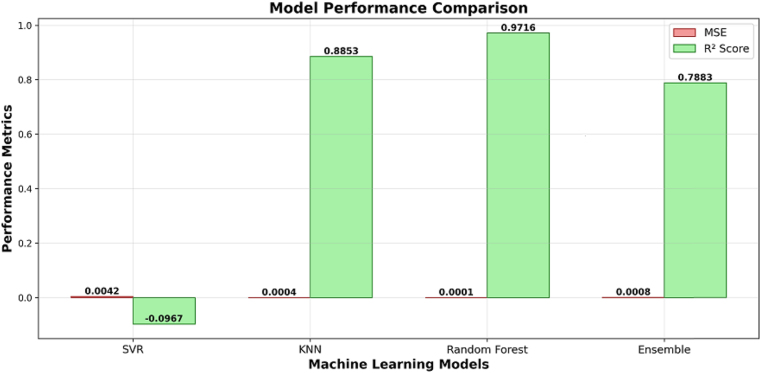
Comparative analysis chart of different machine learning models (SVR, KNN, RF, and Ensemble) using mean square error (MSE) and R^2^ score as evaluation criteria.

**Fig. 4:**
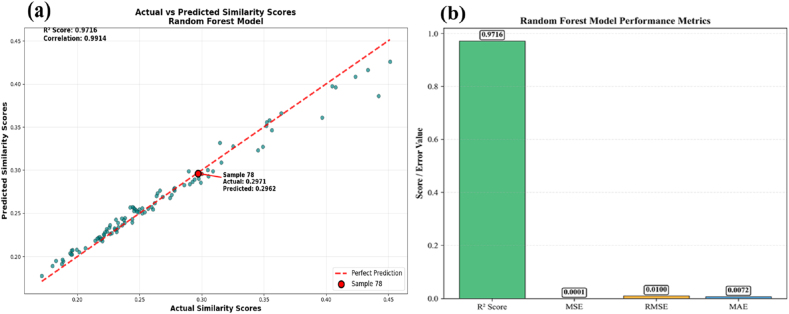
Prediction performance of the random forest model (a) Scatter plot of actual versus predicted similarity scores from the random forest model. (b) Performance evaluation of the random forest regression model. Key performance metrics include R^2^, mean squared error (MSE), root mean squared error (RMSE), and mean absolute error (MAE).

The comparative performance of the implemented machine learning algorithms, including SVR, KNN, Random Forest (RF), and the Ensemble model, is summarized in Fig. 3. Among these models, RF demonstrated significantly higher predictive accuracy, achieving an R^2^ of 0.9716 and the lowest MSE of 0.0001, followed by KNN with an R^2^ of 0.8853. The SVR model showed poor performance with a negative R^2^ value (−0.0967), indicating its limited generalization capability. The Ensemble model provided relatively consistent results but did not surpass the performance of RF. Fig. 4(a) illustrates the correlation between actual and predicted similarity scores using the RF model. The data points align closely along the ideal prediction line (correlation = 0.9914), demonstrating the high agreement and reliability of the model's predictions. A representative compound (Sample 78) displayed nearly identical predicted and actual values (0.2962 vs. 0.2971), confirming the precision of RF in capturing descriptor similarity relationships.

The performance metrics presented in Fig. 4(b) further highlight the statistical robustness of RF (MSE = 0.0001, RMSE = 0.010, MAE = 0.0072). The low error values and high R^2^ support the excellent fit of the model and its minimal tendency toward overfitting. The superior performance of RF is attributed to its decision-tree–based ensemble framework, which efficiently learns complex nonlinear interactions among molecular descriptors and reduces prediction variance. As noted, this ensemble nature provides high stability, enabling the model to capture intricate molecular descriptor patterns while minimizing overfitting an advantage that explains its dominance over the other models (Khamis et al., 2015; Aldakheel et al., 2025). Furthermore, feature-importance analysis in the RF model showed that descriptors associated with molecular size, charge distribution, and hydrophobicity played major roles in similarity prediction. These features are also influential in ligand–protein interactions during docking and in structural stability during molecular dynamics simulations. Additionally, the implementation of 5-fold cross-validation confirmed the stability of these results. The RF model maintained consistently high R^2^ scores and low error metrics across all validation folds, demonstrating minimal variance. This rigorous cross-validation process ensures that the remarkable accuracy (R^2^ = 0.9716) is a true reflection of the model's ability to generalize complex non-linear descriptor relationships, rather than an artifact of overfitting on a limited sample size.

### 3.2 Molecular docking and compounds shortlisting

**Fig. 5:**
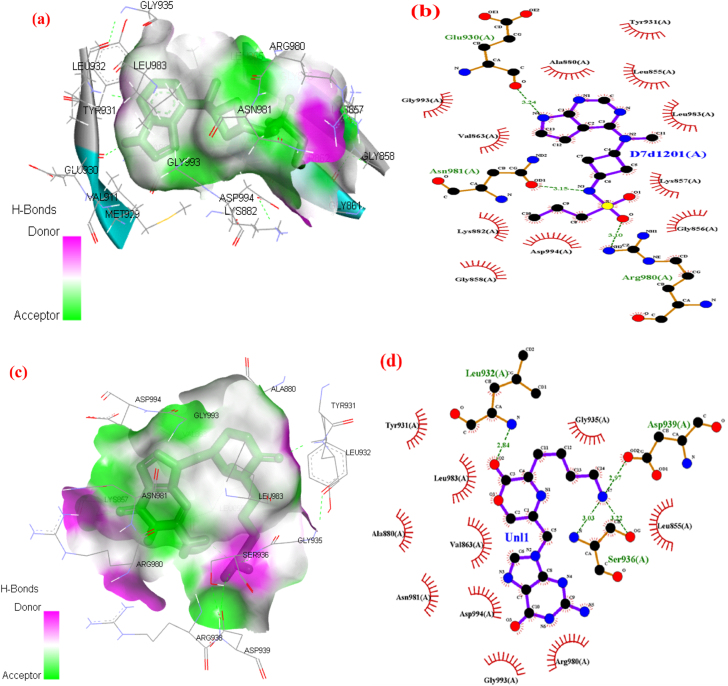
Interaction between the protein–ligand complex. (**a**) three-dimensional binding mode of the ABR and protein complex; (**b**) two-dimensional binding mode of the ABR and protein complex; (**c**) three-dimensional binding mode of the AMO and protein complex; (**d**) two-dimensional binding mode of the AMO and protein complex.

Molecular docking was used to identify the protein's active site and determine the optimal orientation of the ligand-protein complex. Initially, the crystal structure of the protein of all five drugs of the Jak kinase inhibitor class with the above-mentioned protein code was downloaded in pdb format from the (https://www.rcsb.org/). After removing water molecules and separating the protein and ligand using Discovery Studio 2021 (Aguilar-Carrillo et al., 2024), the complex was imported into AutoDock Vina for docking analysis. To prepare the proteins for docking, Coleman charges, Gasteiger charges, and AutoDock atom types were assigned, and a grid box for each drug-protein complex was generated. It was also prepared for the AMO candidate that was screened and selected from the previous step. Then, all proteins, drugs, and AMO were saved in the pdbqt format in specific folders. After molecular docking was performed for all five drugs mentioned above, AMO was run via the command line, and the binding energies of each were calculated and compared. By comparing the physical properties, active site, and binding energy of AMO with the protein of all five drugs mentioned, and considering the highest overlay and the most effective protein-ligand interactions in the active site, ABR with the protein code (PDB ID: 6BBV) was selected as the drug to be compared. The active sites of the proteins AMO and ABR contain ASP 939, Leu 932, and Ser 936, and ABR has Glu930, Arg 980, and Asn 981, all of which are characterized by hydrogen bonds. Both AMO and ABR share overlapping amino acids (Leu 855, Leu 983, ASP 994, Val 863, Gly 993) and exhibit similar functional effects in the protein's active site, suggesting AMO as a potential alternative to ABR for Jak enzyme inhibition. Fig. 5 shows the 2D and 3D protein-AMO and protein-ABR interactions in the active site of the protein after molecular docking. Binding efficiency metrics, including inhibition constant (Pandey and Verma, 2022) (Kᵢ) and ligand efficiency (Edache et al., 2023) (LE), are of particular importance in medicinal chemistry, drug discovery, and drug design. They are used to evaluate the binding efficacy of a ligand (usually a potential drug molecule) to its biological target (such as a protein or enzyme). The inhibition constant (Kᵢ) is a measure of the potency of an enzyme inhibitor. This value is calculated as K_i_ = exp(ΔG/(R×T)), where ΔG is the free energy of binding, R is the gas constant (1.987 cal K^−1^ mol^−1^), and T is the temperature (298.15 K). According to this equation, the lower the K_i_, the less the ligand can inhibit the enzyme. Ligand efficiency (LE) is another binding efficiency metric and is usually expressed per heavy atom (non-hydrogen atom). This value is calculated as LE = -ΔG/N, where ΔG is the binding free energy, and N is the number of heavy atoms (non-hydrogen atoms). Table 2 shows the binding energies for AMO, which has the strongest binding energy of −8.4 kcal/mol, and ABR, with −8.1 kcal/mol, calculated by AutoDock Vina. The binding efficiency metric, including inhibition constant (K_i_) and ligand efficiency (LE) values normalized by the number of heavy atoms, is also reported for both compounds. As shown, AMO has a lower inhibition constant (K_i_) than ABR, suggesting greater inhibitory efficiency against the Jak enzyme.

**Table 2:** Protein active site affinity calculated by AutoDock Vina as well as binding efficiency metrics, including inhibition constant (K_i_) and ligand efficiency (LE) values normalized by the number of heavy atoms for both AMO and ABR compounds.

Structure	Affinity (kcal/mol)	inhibition constant (K_i_)	Ligand efficiency (LE)
**AMO**	−8.4	6.56 × 10^−7^	0.35
**ABR**	−8.1	1.09 × 10^−6^	0.36

### 3.3 ADMET prediction & Pre-Tox analysis

Since the bioavailability of a drug depends on its safety and efficacy, interactions with target biological macromolecules may produce a desirable or undesirable pharmacological effect. Therefore, drug design is a step-by-step evaluation process, and lack of safety and efficacy are the leading causes of drug failure, which mainly depend on the ADME properties. Here, we evaluate the ADME properties of both selected compounds using the SwissADME in silico server to assess pharmacokinetic parameters, including lipophilicity, water solubility, and drug affinity. Lipophilicity means the compounds can easily diffuse through the cell membrane. The hydrophilicity of the compounds means they are more soluble in water, allowing them to be easily absorbed and distributed throughout the body. Table 3 presents the pharmacokinetic and physicochemical properties of both compounds. In comparing the two compounds, the results show that the proposed compound obeys all of Lipinski's rules of five, highlighting its potential as a promising candidate for substitution. Since in silico toxicity assessment is an important method before clinical testing of drug candidates, to support further investigation and a more informed selection of the designed drug, its toxicity was evaluated using the ProTox-3.0 web server and compared with that of the drug ABR. For its more accurate evaluation, several toxicological parameters such as acute toxicity, hepatotoxicity, cytotoxicity, carcinogenicity, mutagenicity, and immunotoxicity were used, and the result was obtained based on the predicted median lethal dose LD50 in mg/kg body weight (Morris-Schaffer and McCoy, 2020), which is listed in Tables S2 and S3 for complete information on both compounds. According to the information obtained, since both structures belong to class 4, the LD50 of the AMO structure is 1000 mg/kg, which is much lower than that of ABR (500 mg/kg). It can be predicted that AMO is a better candidate and a more suitable alternative to ABR.

**Table 3:** The data, physical properties, and pharmacokinetics of ABR and AMO were obtained and compared using the SwissADME web server.

Compounds	MW (g/mol)	No. HBA	No. HBD	No. Rot^a^	cLogP	cLogS	Toxicity risk	Molar Refractivity
**ABR**	323.4	5	2	6	1.81	−4.06	None	86.59
**AMO**	335.3	7	4	6	0.83	−2.85	None	90.87

a No. Rot: count of rotatable bonds.

### 3.4 Molecular dynamics simulation

After screening, the molecular docking results were used to perform molecular dynamics simulations to investigate the stability of AMO and ABR. Molecular dynamics simulations model the system at the atomic level and provide extensive analyses of the structural dynamics of protein-ligand complexes in physiological environments, and consider all its components, such as ligands, proteins, and solvent molecules, to be fully flexible. This flexibility allows them to capture the dynamic behavior of molecular interactions and structural changes, such as folding and complex formation. Here, RMSD, RMSF, Rg, DSSP, PCA, and DCCM analyses were used for comparison.

#### 3.4.1 Root mean square deviation (RMSD)

**Fig. 6:**
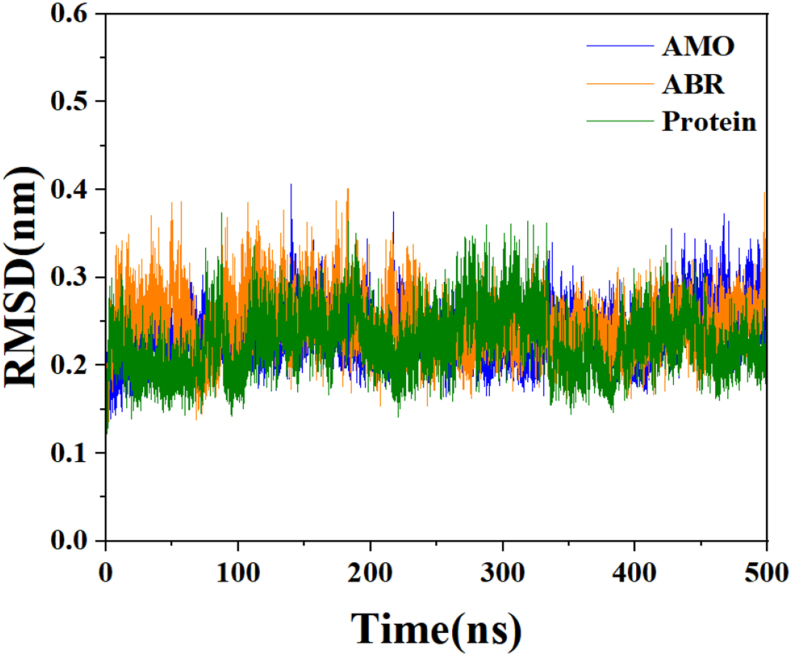
Root mean square deviation (RMSD) plot of protein(green) along with AMO (blue) and ABR (orange) to the protein backbone during the 500 ns MD simulation.

To obtain the equilibrium time of each simulated protein-ligand complex during the molecular dynamics simulation, the root mean square deviation (Samantha et al., 2023) (RMSD) of the AMO, ABR, and protein structures was calculated. Since RMSD plots are commonly used to assess stability and structural changes over simulation time, allowing the system to reach structural equilibrium and estimate simulation duration, one of the most important analyses in molecular dynamics is the comparison of two structures in drug design and discovery. To investigate the stability of the ligand-protein binding state during the simulation, RMSD plots of the protein and both AMO and ABR were calculated relative to the protein backbone as a reference structure. As shown in Fig. 6, the RMSD of the protein (green), which ranges approximately from 0.15 to 0.30 nm, shows little fluctuation, indicating minimal mutations and a high degree of stability. The ABR complex exhibits a wider oscillation range (0.20–0.40 nm) and higher RMSD changes in the early part of the trajectory (0–100 ns). However, the RMSD of the AMO compound is approximately 0.15-0.30 nm, indicating stable binding to the protein over 500 ns, as observed from the initial onset when the protein reaches equilibrium, indicating a stronger and more stable binding of this ligand to the protein than ABR over the simulation time.

#### 3.4.2 Root mean square fluctuations (RMSF)

**Fig. 7:**
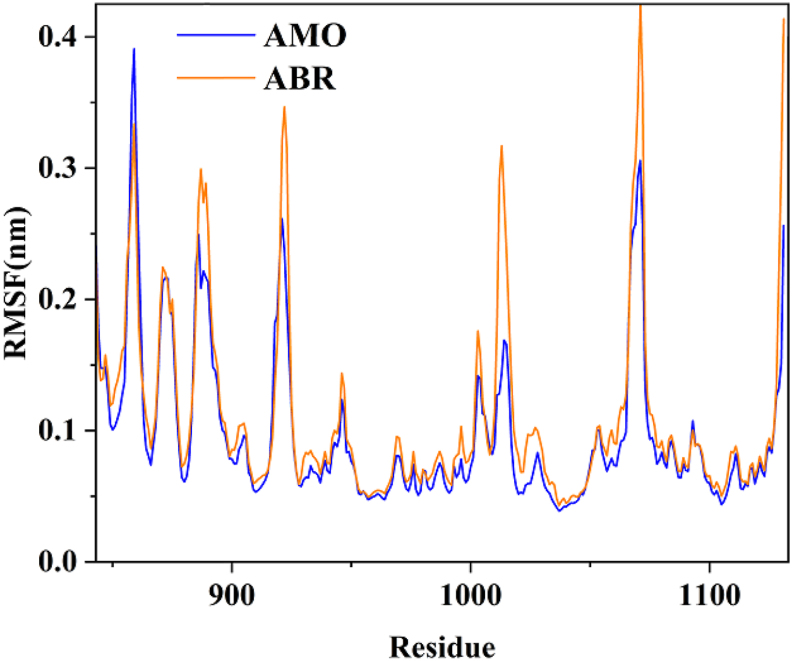
Root mean square fluctuations (RMSF) plot showing residue flexibility comparison between AMO (blue) and ABR (orange) to the protein backbone during simulation.

The root-mean-square fluctuation (Knapp et al., 2011) (RMSF) is the amount of movement of each atom or residue in molecular dynamics simulations. It helps assess atomic flexibility, bond stiffness, and interaction strength, making it a critical descriptor for evaluating and optimizing ligand stability. Low RMSF values indicate that atoms are positioned close to their average positions. The drug is firmly positioned in the active site with minimal structural slippage and stable binding. Still, high RMSF values typically (>0.5 nm) indicate loose mobility or binding, indicating that parts of the drug (e.g., flexible side chains or polar tail groups) are oscillating. As shown in Fig. 7, both ligands exhibit similar RMSF patterns. ABR exhibits slightly higher RMSF values in several regions, particularly prominent peaks around residues 875, 950, 1000, and ∼1110. These regions correspond to flexible loops or terminal segments that are generally more mobile. However, AMO stabilizes the protein structure typically more effectively than ABR. The lower RMSF of AMO across most regions suggests it may contribute to a more stable and possibly functionally favorable protein state compared to ABR. These results complement the RMSD analysis and confirm the more appropriate AMO alternative.

#### 3.4.3 Radius of gyration (R_g_) and hydrogen bond

**Fig. 8:**
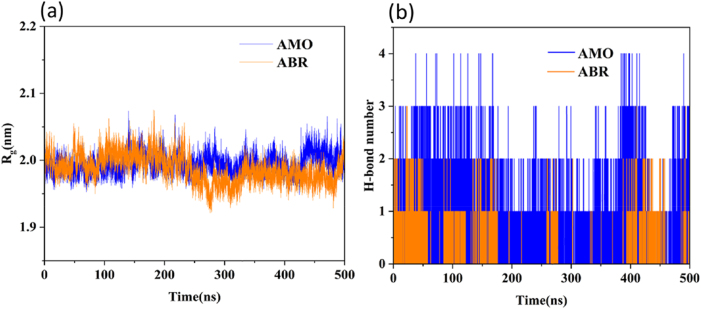
Molecular dynamics simulation inferred findings. (a) Rg plot for the two ligands AMO and ABR (b) Hydrogen bond number during 500 ns simulation run.

The radius of gyration (Rampogu et al., 2022) (Rg) in molecular dynamics is a key measure of a molecule's compactness and structural stability, representing the average distance of its atoms from its center of mass. A lower Rg value during simulation indicates a more rigid, folded, and stable structure, while an increase in Rg can indicate unfolding, flexibility, or structural rearrangement. In drug design and discovery, drug candidates can be compared based on the change in Rg they induce in the target protein. A stable, unchanging Rg profile in simulation indicates a stable, folded complex, which is often favorable for stability. A significant increase in Rg may indicate instability and is generally less desirable. As shown in Fig. 8(a), Rg for ABR is close to 2.0 nm but shows slightly more pronounced fluctuations, especially in the early stages (0–200 ns). There are occasional downward shifts below 2.0 nm, but the occurrence of frequent and larger deviations indicates a relatively less stable and less compact structure. However, Rg for AMO remains around 2.0 nm with slight fluctuations and maintains a relatively constant trend during 500 ns, indicating compactness, less structural change, and high stability compared to AMO.

Hydrogen bond analysis (Halder et al., 2023) is a fundamental step in characterizing and optimizing ligand-protein interactions in drug design and discovery. Hydrogen bonds (H bonds) are critical noncovalent interactions that play an important role in complex stability and can predict ligand affinity and drug success in molecular dynamics (MD) simulations. Fluctuations or decreases in H bonds may indicate ligand instability. H-bond profiles can help identify promising drug candidates by revealing how effectively a ligand binds to its protein target. According to Fig. 8)b), ABR forms fewer hydrogen bonds, mostly fluctuating between 0 and 2, and long periods show only 0 or 1 hydrogen bond. The orange horizontal stripes are thinner and less frequent, indicating fewer and more unstable hydrogen bonds. In contrast, AMO consistently forms more hydrogen bonds, often up to 3-4 during the simulation. The density of the blue lines indicates that these hydrogen bonds are stable and frequent throughout the trajectory. Since hydrogen bonds play a significant role in stabilizing the ligand in the active site. Therefore, AMO forms a more stable complex in the active site of the protein than ABR because it is supported by stronger and more frequent hydrogen bond interactions during the 500 ns simulation.

#### 3.4.4 SASA and DSSP analyses

**Fig. 9:**
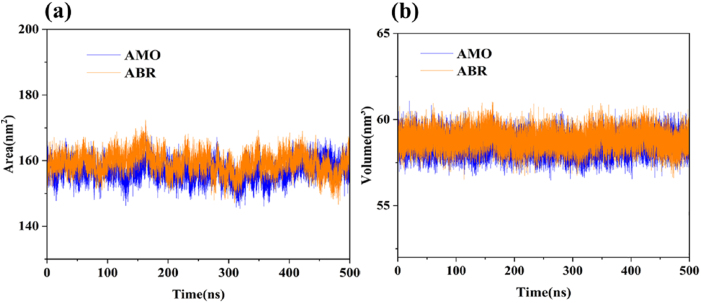
Time-dependent changes in solvent-accessible surface area (a) and volume (b) for AMO and ABR complexes over 500 ns of simulation, showing structural stability and differences in molecular compactness.

Solvent-accessible surface area (SASA) and volume analysis (Wan et al., 2017) are key to understanding how a drug molecule interacts with its environment, typically the target protein and the solvent. It is an important measure for investigating drug solubility, binding site accessibility, and structural changes during simulation. Solvent-accessible area (SAS) was calculated to assess the surface area and solvent-exposed volume of the protein for AMO and ABR. Higher SASA values typically indicate greater solvent exposure, which may affect receptor-ligand interactions and overall structure stability. In Fig. 9(a), the surface SAS values for AMO (blue) and ABR (orange) fluctuate between approximately 140 and 180 nm^2^. Still, the ABR structure consistently exhibits slightly higher surface area values than AMO throughout the trajectory. Both are relatively stable, but ABR is intermittently higher than AMO, indicating that it exposes more solvent accessible surface area during the simulation. As shown in Fig. 9(b), the volume SAS values for both AMO and ABR range from approximately 55 to 62 nm^3^ and remain very stable for more than 500 ns. ABR exhibits slightly higher volume values than AMO, indicating that, like surface SAS, ABR occupies slightly more space and volume, which could be due to a less compact or more dynamic structure. Overall, these findings are consistent with previous structural metrics (RMSD, RMSF, Rg, H-bonds) and reinforce the idea that AMO forms a more stable and compact complex than ABR.

**Fig. 10:**
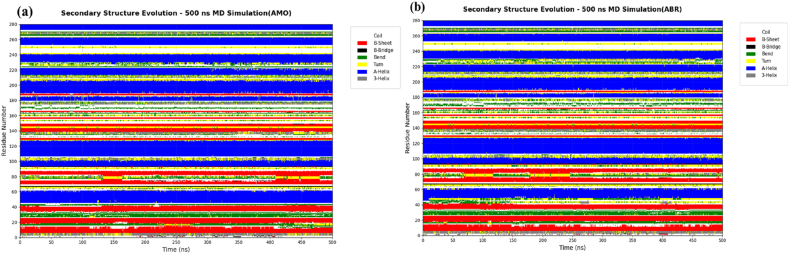
DSSP secondary structure analysis as a function of simulation time for the trajectories of (a) AMO, (b) ABR. The secondary structure type of each residue as a function of simulation time is color coded: black, β-bridge; red, β-sheet; white, coil; green, bend; yellow, turn; blue, α-helix; gray, 3-helix.

DSSP (Hosseini et al., 2025; Olubiyi and Strodel, 2012) (Definition of Secondary Structure of Proteins) is an algorithm based on the atomic coordinates of each residue in a protein that is widely used in molecular dynamics (MD) simulations to track structural changes over time. DSSP evaluates hydrogen bonding patterns and geometric features directly from the atomic coordinates obtained during the MD trajectory and assigns specific secondary structure types, such as α-helix, β-sheet, turn, and others, to each amino acid residue in each frame. In drug design and discovery, DSSP analysis of MD trajectories enables researchers to precisely determine how ligand binding, mutations, or environmental changes affect protein secondary structure, folding stability, and aggregation propensity. As shown in Fig. 10(b), the ABR structure shows regions of stable α-helix with bands, although some helices may be shorter or show subtle instabilities. The β-sheet is clearly visible and stable, although in some regions it may appear slightly discontinuous, suggesting increased dynamics or local unfolding. In contrast, in Fig. 10(a), the AMO structure shows continuous blue bands for specific regions of the remaining α-helix, indicating a more ordered and stable conformation. The β-sheet content is stably preserved, and the secondary structure elements (especially the helices and sheets) are largely stable over the 500 ns simulation. However, both retain their essential secondary structure motifs (α-helices and β-sheets) during the long MD simulation, indicating high intrinsic stability. But in ABR, subtle differences in the connectivity, length, and flexibility of secondary structure elements may reflect sequence, mutation, or environmental influences that can affect protein stability.

#### 3.4.5 Principal component analysis (PCA)

**Fig. 11:**
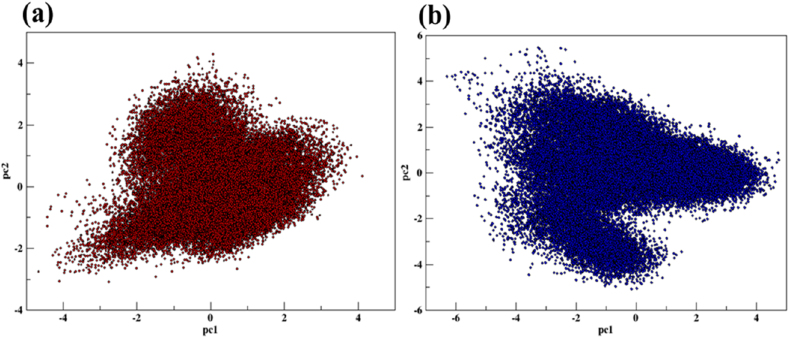
Principal component analysis (PCA) on the Two-dimensional conformational projections of MD trajectories of protein-ligand complexes. (a)AMO. (b) ABR.

Principal component analysis (Nemaysh and Luthra, 2017; Aldakhil and Altharawi, 2025) (PCA) is a powerful technique for analyzing structural dynamics, observed complex atomic fluctuations, and collective motions in protein-ligand systems. It often shows that ligand binding can restrict or stabilize certain protein motions. Here, we evaluated and compared the large-scale motions of the proteins using essential dynamics (ED) by PCA analysis for both AMO and ABR to confirm the MD simulation results further. In Fig. 11, the scatter plots of the first two principal components (PC1 and PC2) for the protein-ligand complexes AMO (a) and ABR (b) show the necessary structural sampling during molecular dynamics. The ABR PCA plot shows a broader, more diffuse distribution along both PC1 and PC2. This broader distribution indicates increased structural flexibility, with the complex accessing more regions in the required subspace. In contrast, the AMO PCA plot shows a relatively tight, dense distribution, indicating that complex samples occupy a more restricted structural space. Most points are clustered in the central region and show less dispersion along the PC1 and PC2 axes than the ABR. This suggests less dominant, large-scale motions and possibly greater stability or rigidity in the AMO structure under simulation, confirming the analyses described above.

#### 3.4.6 Dynamic cross-correlation matrix (DCCM) analysis

**Fig. 12:**
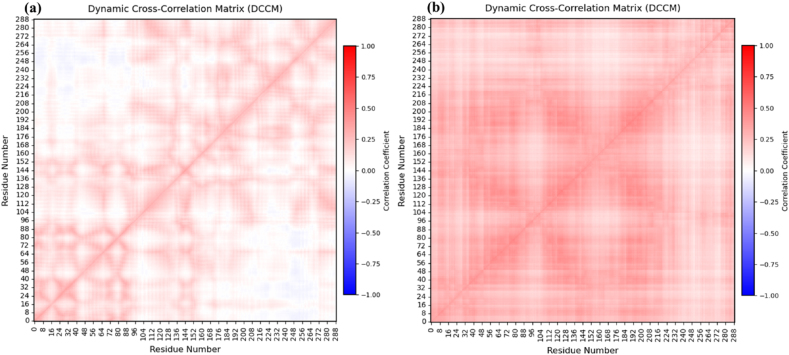
The dynamic cross-correlation matrix (DCCM) of backbone (a) AMO(b) ABR contains contours of different colors. Correlated motion and anti-correlated motions are represented by red and light blue color contours, respectively.

DCCM (Dynamic Cross-Correlation Matrix)(Rahimi et al., 2023; Upadhyay, 2014) analysis is commonly used in molecular dynamics (MD) simulations, especially for protein-ligand systems. It measures the degree of correlation (moving together, shown in red) or anti-correlation (moving in the opposite direction, shown in blue). In protein-ligand systems, DCCM can reveal the effects of stabilization or changes in domain motion upon ligand binding, which involves evaluating strong (red) or weak/absent (white/blue) correlation patterns. DCCM analysis is a logical approach for visualizing and quantifying dynamic association and stabilization in protein-ligand complexes. As shown in Fig. 12, for both patterns, a similar correlation matrix is observed, with the points of correlation appearing as red spots. Fig. 12(b) shows a more homogeneous pattern in ABR, with most regions moving together, indicating a more coordinated overall dynamics, less flexibility, and fewer opposing amplitude movements. Fig. 12(a) shows in AMO that the whiter, brighter regions indicate dynamic, active, random movements, and the red color remains dominant, indicating more coordinated, uniform, and stable movements in this system.

### 3.5 MM-PBSA analysis

**Fig. 13:**
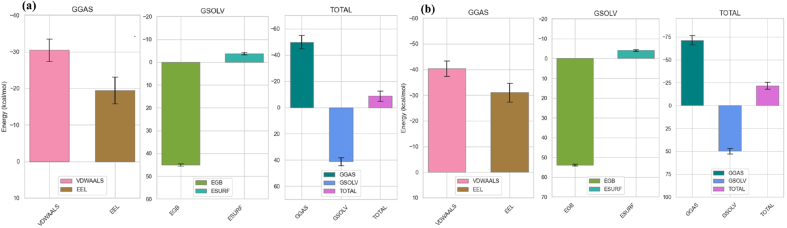
MM-PBSA (Molecular Mechanics Poisson-Boltzmann Surface Area) plot showing the free energy of (a) protein-AMO and (b) protein-ABR binding. ΔE_vdW_: Van der Waals energy contribution; ΔE_ele_: Electrostatic interactions in a vacuum; ΔE_GB_: the electrostatic contribution to the solvation free energy calculated by PB; ΔE_SURF_: Nonpolar solvation energy calculated by an empirical model; ΔG_GAS_: Total gas-phase energy (ΔE_vdW_ + ΔEele); ΔG_SOLV_: Total solvation energy (ΔE_GB_ + ΔE_SURF_).

The MM-PBSA approach is a measure of the energy released upon ligand binding in molecular dynamics simulations. Although the MM-GBSA method is computationally fast, it relies on approximations that can reduce accuracy. The MM-PBSA method solves the PB equation, providing a more accurate and reliable estimate of binding interactions, and is very important in molecular dynamics simulations, where it enhances sensitivity and accuracy. we analyzed the binding free energy of AMO and ABR complexes by performing MM-PBSA calculations. Fig. 13 shows the MM-PBSA results for the binding free energy of both protein-AMO and protein-ABR. Energy (G_GAS_: van der Waals and electrostatic), solvation energy (G_SOLV_: polar and nonpolar solvation), and total binding energy (TOTAL). This plot shows the noncovalent energies (G_GAS_), solvation (G_SOLV_), and total energies to determine the net stability of the protein-ligand complex. G_Gas_ is the sum of van der Waals and electrostatic energies, G_SOLV_ is the sum of solvation energies, and Total is the final and net binding free energy after addition of G_GAS_ and G_SOLV_. As shown in Fig. 13(b), G_Gas_ the sum of van der Waals and electrostatic energies is strongly negative (∼-71 kcal/mol), and G_SOLV_ is strongly positive (∼+65 kcal/mol), which is unfavorable. And the total energy is negative, which is favorable overall binding. In contrast, Fig. 13(a) shows that the protein-AMO binding is primarily stabilized by strong van der Waals and electrostatic attractions (G_GAS_). Also, the negative total energy of −8.74 is a negative binding energy, which is thermodynamically favorable, indicating the thermodynamic stability of the protein-ligand binding.

## 4 Conclusion

In this research, we designed structures based on L-amino acids and nucleotide bases and compared them across a wide range of drugs that inhibit the JAK kinase enzyme. Then, molecular mechanics and frequency optimization were performed using Spartan, and the absence of imaginary frequencies was confirmed. The physical properties of the drugs and proposed structures were obtained. Next, 5 JAK kinase inhibitor drugs and 100 proposed structures were screened using machine learning models (SVR, RF, KNN, Ensemble). However, the small dataset used in this study limits the generalizability of the models and necessitates more rigorous validation with larger datasets in future research. Molecular docking was performed at the most effective active site of the protein to identify the best drug. The drug ABR and the AMO structure with the best compatibility were selected. For further investigation, molecular dynamics (MD) simulations were performed for both AMO and ABR. The RMSD and RMSF plots indicate that AMO has better dynamic stability than ABR. Rg and hydrogen-bond analysis revealed stronger protein-AMO interactions. In addition, protein secondary structure analysis using the DSSP algorithm showed that AMO was more stable than ABR. The PCA plot for AMO showed a relatively compact, dense distribution compared to ABR. The pharmacokinetic properties of AMO and ABR were calculated and compared. The toxicity effects were calculated using the ProTox-3.0 web server. Since both structures belong to class 4, the AMO structure showed much lower toxicity than ABR, suggesting that AMO is a safer candidate and a more suitable alternative to ABR. Finally, MM-PBSA results showed that the binding energy is negative, indicating thermodynamic favorability, and that the AMO-protein binding energy is strong. Based on the computational results, this alternative candidate is expected to be a suitable option for future research and therapeutic applications due to the use of natural starting materials in its structure. Further experimental (laboratory) evaluations and clinical trials are required to confirm its efficacy and therapeutic potential.

## CrediT author statement

Morteza Hosseini: performed the project and wrote the main manuscript. Alireza Fattahi conducted the project and revised the manuscript.

## Declaration of competing interest

The authors declare that they have no known competing financial interests or personal relationships that could have appeared to influence the work reported in this paper.

## Data Availability

All data generated or analyzed during this study are included in this published article [and its supplementary information files].
