# Supplementary material for: Nucleo-amino acid derived AMO as a potential JAK inhibitor: machine learning screening, docking, molecular dynamics, ADMET/toxicity, and MM-PBSA analysis
Source: Curr Res Struct Biol. 2026 Jun 15;12:100191. doi: 10.1016/j.crstbi.2026.100191 (PMC13315765; doi:10.1016/j.crstbi.2026.100191)
Supplement: Multimedia component 1 [file mmc1.docx]

**"** **Natural product–derived AMO as a potential JAK inhibitor: machine-learning screening, docking, molecular dynamics, ADMET/toxicity, and MM-PBSA analysis"**

Morteza Hosseini,^1^ Alireza Fattahi^1*^

^1^Department of Chemistry

Institute for Convergence Science and Technology, Bioinformatics Group

Sharif University of Technology, Tehran, Iran

*Corresponding author. [**fattahi@sharif.edu**](mailto:fattahi@sharif.edu)

**Contents:**

[S1. Molecular docking……………………………](#_Toc190809588)………………..…………….….….….2

[S2. The structural model ..…................................................................................](#_Toc190809589).......5

S3. Pre-Tox analyses………...…………………………………………….….…...15

# **S1. Molecular docking**

The **input** file prepared at the docking step for the **molecular dynamics simulation** step.

**AMO**

**lig_ini.gro**

**45**

**1LIG H1 1 -0.554 0.296 1.147**

**1LIG N1 2 -0.504 0.298 1.059**

**1LIG C1 3 -0.452 0.163 1.027**

**1LIG C2 4 -0.349 0.124 1.134**

**1LIG O1 5 -0.247 0.223 1.157**

**1LIG C3 6 -0.285 0.353 1.167**

**1LIG O2 7 -0.224 0.431 1.240**

**1LIG C4 8 -0.394 0.397 1.068**

**1LIG C5 9 -0.395 0.149 0.884**

**1LIG N2 10 -0.497 0.112 0.788**

**1LIG C6 11 -0.522 -0.015 0.742**

**1LIG N3 12 -0.617 -0.016 0.650**

**1LIG C7 13 -0.654 0.115 0.638**

**1LIG C8 14 -0.583 0.196 0.723**

**1LIG N4 15 -0.592 0.331 0.739**

**1LIG C9 16 -0.681 0.386 0.661**

**1LIG N5 17 -0.702 0.519 0.665**

**1LIG N6 18 -0.758 0.315 0.571**

**1LIG C10 19 -0.752 0.178 0.552**

**1LIG O3 20 -0.822 0.120 0.469**

**1LIG H2 21 -0.662 0.562 0.748**

**1LIG H3 22 -0.796 0.552 0.646**

**1LIG H4 23 -0.822 0.363 0.510**

**1LIG C11 24 -0.454 0.535 1.104**

**1LIG C12 25 -0.555 0.583 0.999**

**1LIG C13 26 -0.617 0.717 1.037**

**1LIG C14 27 -0.701 0.772 0.922**

**1LIG N7 28 -0.621 0.786 0.802**

**1LIG H5 29 -0.552 0.861 0.815**

**1LIG H6 30 -0.680 0.817 0.725**

**1LIG H7 31 -0.535 0.095 1.030**

**1LIG H8 32 -0.401 0.107 1.226**

**1LIG H9 33 -0.299 0.036 1.097**

**1LIG H10 34 -0.346 0.404 0.973**

**1LIG H11 35 -0.319 0.074 0.885**

**1LIG H12 36 -0.356 0.245 0.855**

**1LIG H13 37 -0.470 -0.102 0.775**

**1LIG H14 38 -0.374 0.606 1.110**

**1LIG H15 39 -0.504 0.525 1.198**

**1LIG H16 40 -0.633 0.510 0.991**

**1LIG H17 41 -0.504 0.595 0.906**

**1LIG H18 42 -0.538 0.787 1.059**

**1LIG H19 43 -0.679 0.704 1.122**

**1LIG H20 44 -0.741 0.868 0.950**

**1LIG H21 45 -0.781 0.704 0.902**

**-------------------------------------------------------------------------------------------------------------------**

**ABR**

**lig_ini.gro**

**45**

**1LIG C1 1 -0.530 0.383 0.775**

**1LIG N1 2 -0.517 0.275 0.875**

**1LIG C2 3 -0.293 0.251 1.172**

**1LIG C3 4 -0.364 0.204 1.057**

**1LIG C4 5 -0.322 0.069 1.036**

**1LIG C5 6 -0.230 0.038 1.134**

**1LIG C6 7 -0.449 0.299 0.993**

**1LIG N2 8 -0.462 0.425 1.046**

**1LIG N3 9 -0.211 0.149 1.217**

**1LIG N4 10 -0.303 0.377 1.227**

**1LIG C7 11 -0.389 0.455 1.157**

**1LIG H1 12 -0.150 0.154 1.297**

**1LIG H2 13 -0.253 0.407 1.306**

**1LIG C8 14 -0.587 0.150 0.853**

**1LIG C9 15 -0.718 0.149 0.769**

**1LIG C10 16 -0.667 0.017 0.703**

**1LIG C11 17 -0.523 0.050 0.752**

**1LIG N5 18 -0.690 -0.001 0.561**

**1LIG H3 19 -0.615 0.032 0.501**

**1LIG O1 20 -0.918 -0.072 0.625**

**1LIG S 21 -0.835 -0.059 0.511**

**1LIG O2 22 -0.877 0.022 0.402**

**1LIG C12 23 -0.798 -0.213 0.452**

**1LIG C13 24 -0.856 -0.324 0.542**

**1LIG C14 25 -0.880 -0.453 0.465**

**1LIG H4 26 -0.584 0.347 0.691**

**1LIG H5 27 -0.581 0.466 0.819**

**1LIG H6 28 -0.432 0.414 0.744**

**1LIG H7 29 -0.313 0.343 1.223**

**1LIG H8 30 -0.356 0.004 0.958**

**1LIG H9 31 -0.182 -0.057 1.145**

**1LIG H10 32 -0.401 0.554 1.195**

**1LIG H11 33 -0.592 0.130 0.958**

**1LIG H12 34 -0.728 0.231 0.701**

**1LIG H13 35 -0.814 0.155 0.815**

**1LIG H14 36 -0.711 -0.078 0.728**

**1LIG H15 37 -0.471 -0.032 0.797**

**1LIG H16 38 -0.447 0.081 0.683**

**1LIG H17 39 -0.839 -0.224 0.353**

**1LIG H18 40 -0.692 -0.224 0.451**

**1LIG H19 41 -0.787 -0.343 0.621**

**1LIG H20 42 -0.950 -0.290 0.579**

**1LIG H21 43 -0.787 -0.489 0.427**

**1LIG H22 44 -0.923 -0.526 0.531**

**1LIG H23 45 -0.948 -0.435 0.384**

**---------------------------------------------------------------------------------------------------------------------**

**S2. The structural model**

**Table S1*.*** Proposed structural properties for designed structures based on L-amino acids and nucleotide bases

| **Entry** | **Structure** | **Volume(Å^3^)** | **Area (Å^2^)** | **Polarizability** | **PSA^*^(Å^2^)** |
| --- | --- | --- | --- | --- | --- |
| 1 | 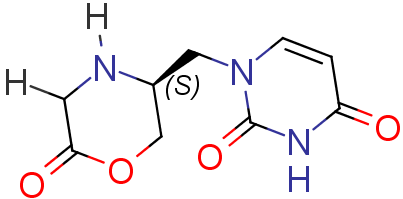 | 200.98 | 211.78 | 54.78 | 67.62 |
| 2 | 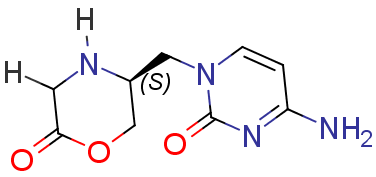 | 208.79 | 233.16 | 55.45 | 80.10 |
| 3 | 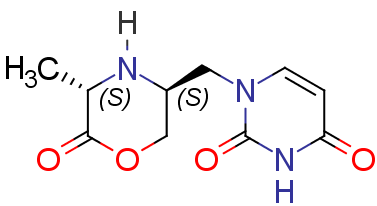 | 223.82 | 247.40 | 56.57 | 73.65 |
| 4 | 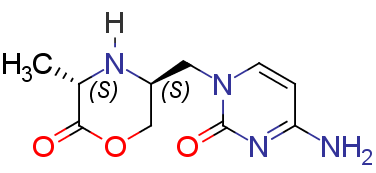 | 227.03 | 251.67 | 56.93 | 79.40 |
| 5 | 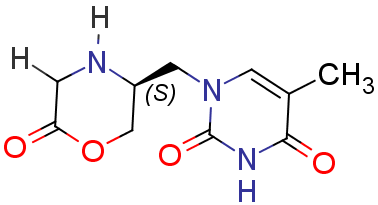 | 223.58 | 246.67 | 56.60 | 74.32 |
| 6 | 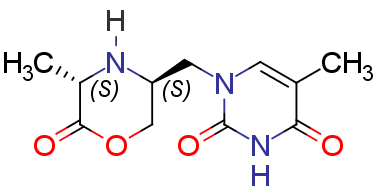 | 242.12 | 266.97 | 58.13 | 73.31 |
| 7 | 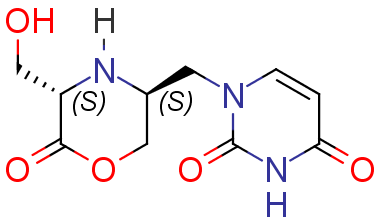 | 231.16 | 255.64 | 57.16 | 92.40 |
| 8 | 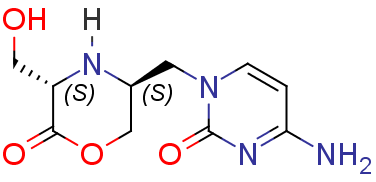 | 234.39 | 259.99 | 57.53 | 97.94 |
| 9 | 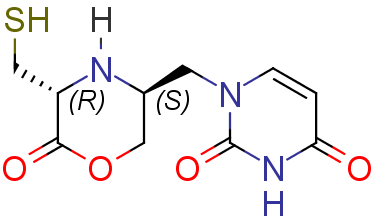 | 241.34 | 266.43 | 57.99 | 72.47 |
| 10 | 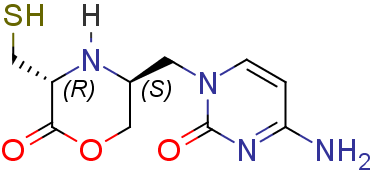 | 243.62 | 266.83 | 58.38 | 73.47 |
| 11 | 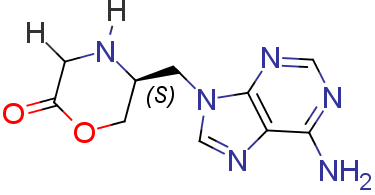 | 228.13 | 253.12 | 57.13 | 83.39 |
| 12 | 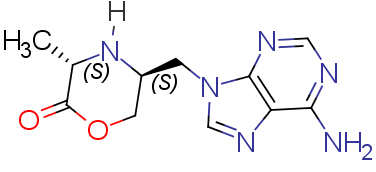 | 246.36 | 271.74 | 58.61 | 82.76 |
| 13 | 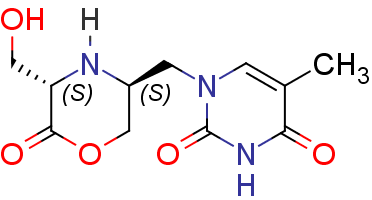 | 249.29 | 275.05 | 58.69 | 91.54 |
| 14 | 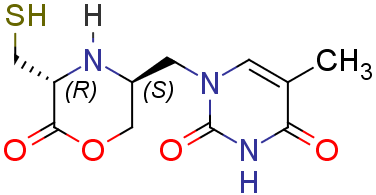 | 259.46 | 285.81 | 59.51 | 71.94 |
| 15 | 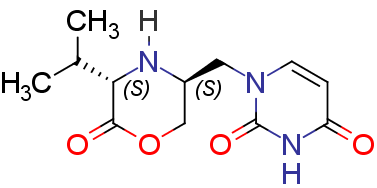 | 260.32 | 283.45 | 59.53 | 72.43 |
| 16 | 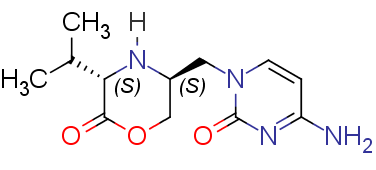 | 263.51 | 287.72 | 59.89 | 78.23 |
| 17 | 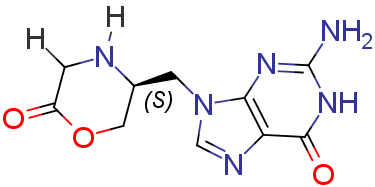 | 234.91 | 261.88 | 57.67 | 101.56 |
| 18 | 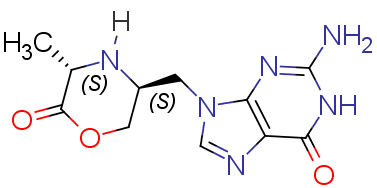 | 253.14 | 280.51 | 59.15 | 101.0 |
| 19 | 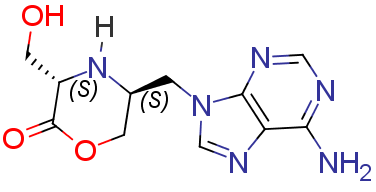 | 253.70 | 279.99 | 59.20 | 102.0 |
| 20 | 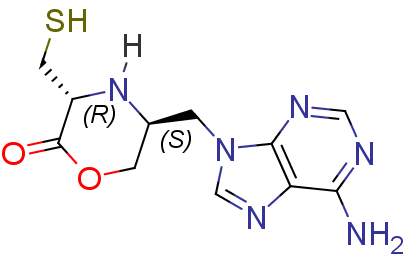 | 263.89 | 288.20 | 60.04 | 82.29 |
| 21 | 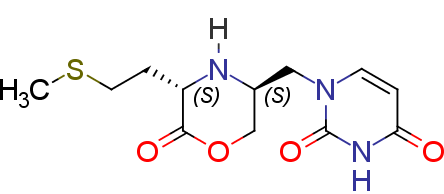 | 274.49 | 287.28 | 60.74 | 64.16 |
| 22 | 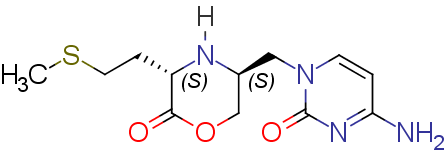 | 279.34 | 303.21 | 61.33 | 73.26 |
| 23 | 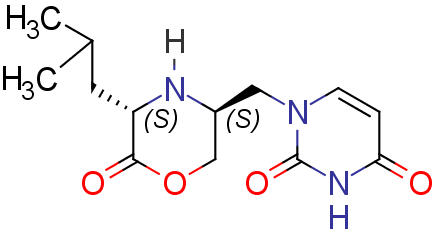 | 273.59 | 285.56 | 60.67 | 67.08 |
| 24 | 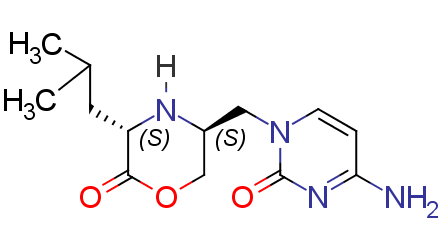 | 279.09 | 303.83 | 61.30 | 77.03 |
| 25 | 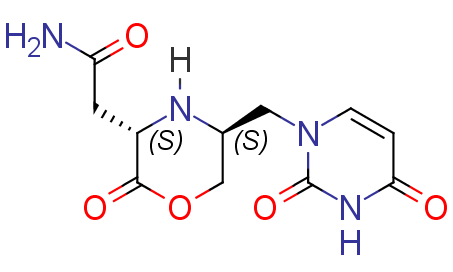 | 250.01 | 266.79 | 58.74 | 99.57 |
| 26 | 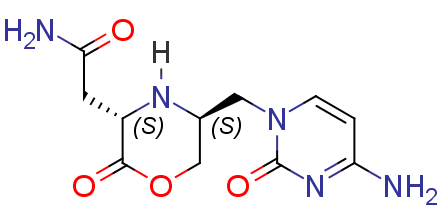 | 255.69 | 283.46 | 59.39 | 115.97 |
| 27 | 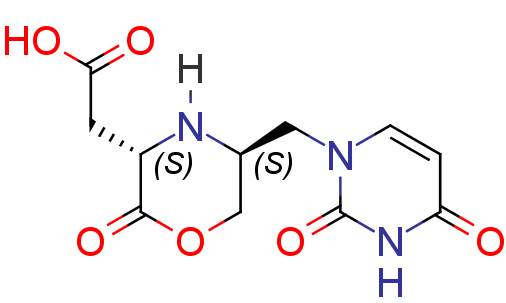 | 247.06 | 262.73 | 58.50 | 95.11 |
| 28 | 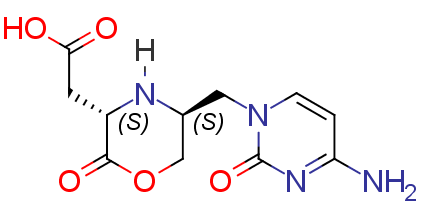 | 252.38 | 280.49 | 59.14 | 112.40 |
| 29 | 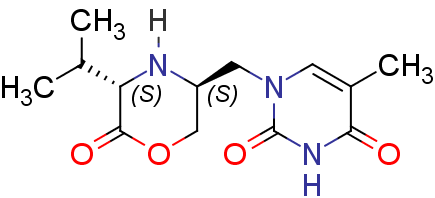 | 273.33 | 284.53 | 60.73 | 65.42 |
| 30 | 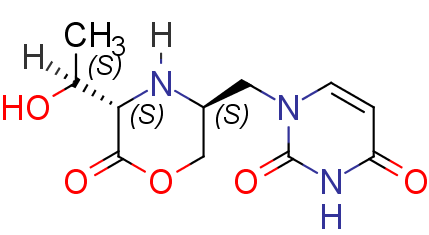 | 244.84 | 254.07 | 58.33 | 82.39 |
| 31 | 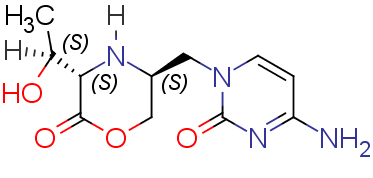 | 250.49 | 275.78 | 58.96 | 96.60 |
| 32 | 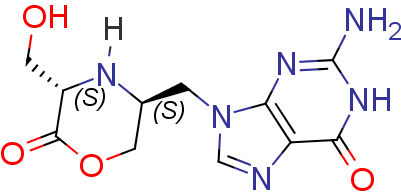 | 257.50 | 273.95 | 59.63 | 115.86 |
| 33 | 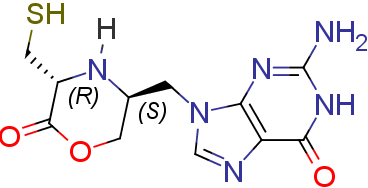 | 267.82 | 286.57 | 60.52 | 96.34 |
| 34 | 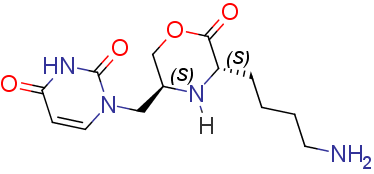 | 284.59 | 303.89 | 61.54 | 85.12 |
| 35 | 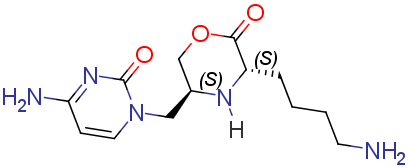 | 290.54 | 321.52 | 62.24 | 102.90 |
| 36 | 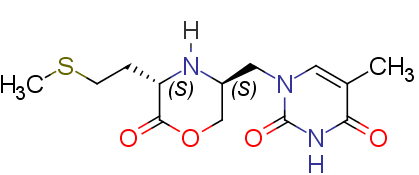 | 292.64 | 306.73 | 62.30 | 63.65 |
| 37 | 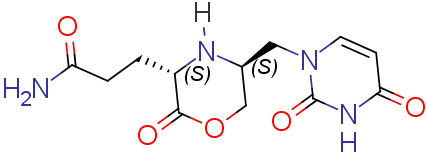 | 272.36 | 295.95 | 60.51 | 106.09 |
| 38 | 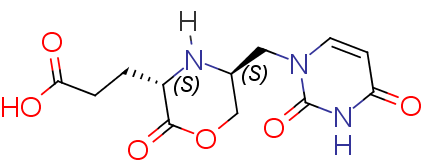 | 264.98 | 279.65 | 59.95 | 93.16 |
| 39 | 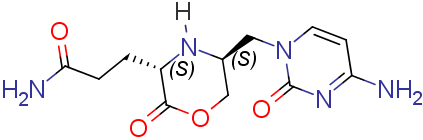 | 273.64 | 302.05 | 60.93 | 116.44 |
| 40 | 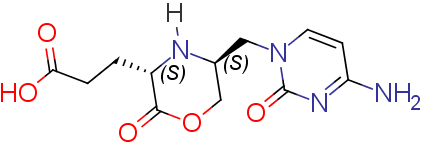 | 273.27 | 299.49 | 60.67 | 111.49 |
| 41 | 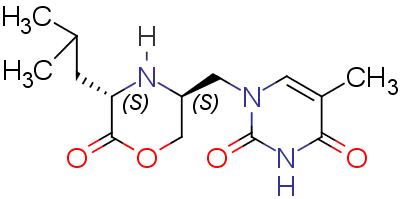 | 291.75 | 305.02 | 62.23 | 66.58 |
| 42 | 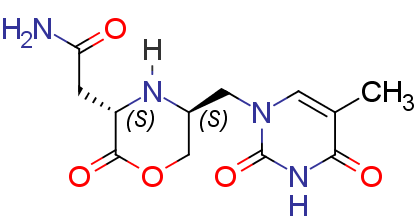 | 268.15 | 286.31 | 60.30 | 99.03 |
| 43 | 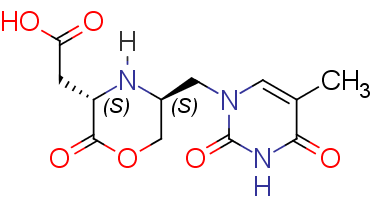 | 265.20 | 282.20 | 60.06 | 94.53 |
| 44 | 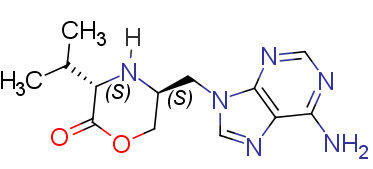 | 282.84 | 307.78 | 61.57 | 81.24 |
| 45 | 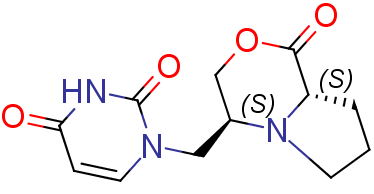 | 274.19 | 280.83 | 60.70 | 77.97 |
| 46 | 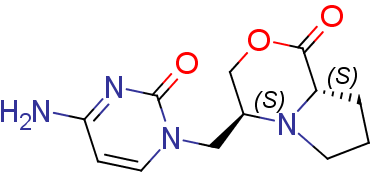 | 279.16 | 299.96 | 61.33 | 87.67 |
| 47 | 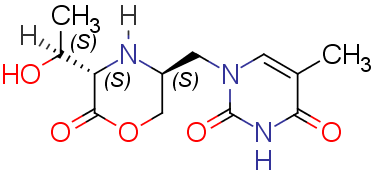 | 267.55 | 292.08 | 60.17 | 89.48 |
| 48 | 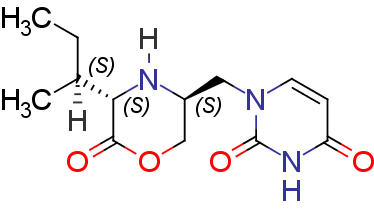 | 273.46 | 283.50 | 60.64 | 65.48 |
| 49 | 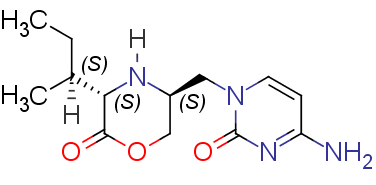 | 279.38 | 306.32 | 61.34 | 77.21 |
| 50 | 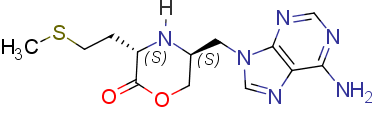 | 302.62 | 331.45 | 63.17 | 81.63 |
| 51 | 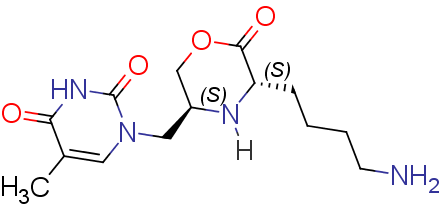 | 302.74 | 323.81 | 63.09 | 85.56 |
| 52 | 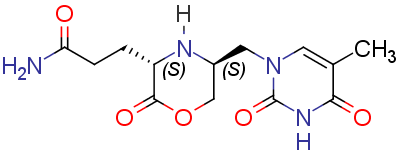 | 286.14 | 303.73 | 61.76 | 97.57 |
| 53 | 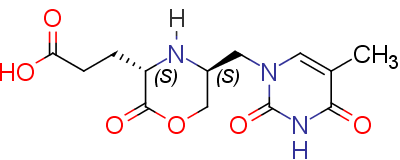 | 283.14 | 299.17 | 61.51 | 92.64 |
| 54 | 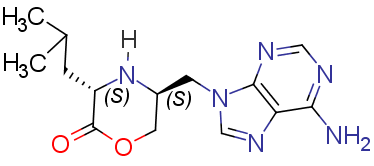 | 301.17 | 328.73 | 63.05 | 82.24 |
| 55 | 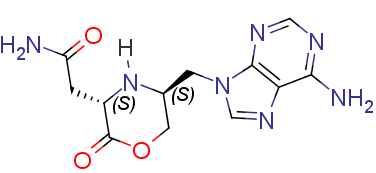 | 277.06 | 300.88 | 61.10 | 116.54 |
| 56 | 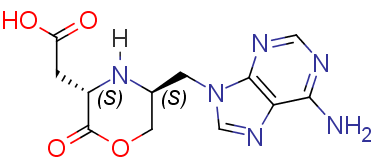 | 271.90 | 292.50 | 60.68 | 114.54 |
| 57 | 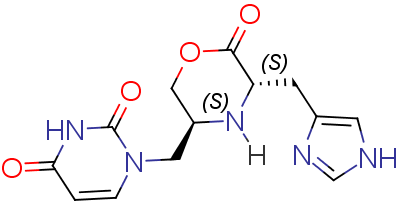 | 277.31 | 290.23 | 60.95 | 79.88 |
| 58 | 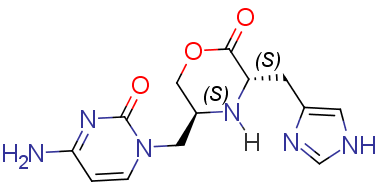 | 282.41 | 306.64 | 61.58 | 93.93 |
| 59 | 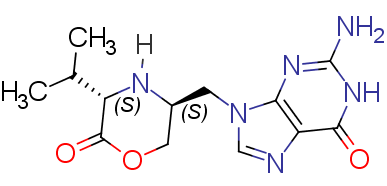 | 286.36 | 301.40 | 61.98 | 96.46 |
| 60 | 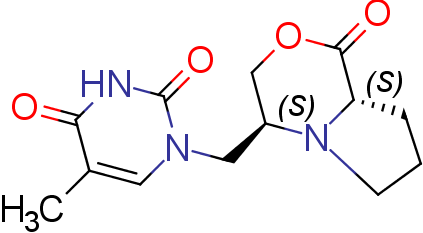 | 267.46 | 286.15 | 60.16 | 63.39 |
| 61 | 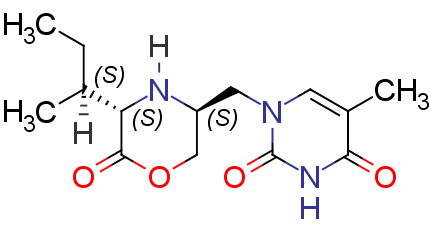 | 272.18 | 299.07 | 60.72 | 99.89 |
| 62 | 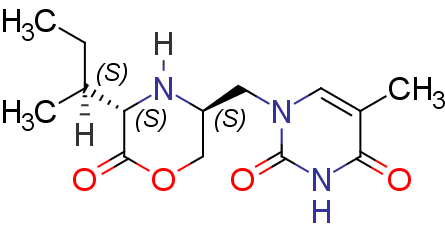 | 291.61 | 302.95 | 62.21 | 65.35 |
| 63 | 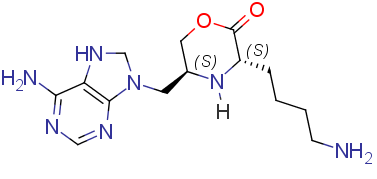 | 316.09 | 346.89 | 64.22 | 73.30 |
| 64 | 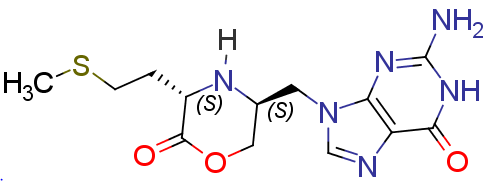 | 306.99 | 333.27 | 63.69 | 96.37 |
| 65 | 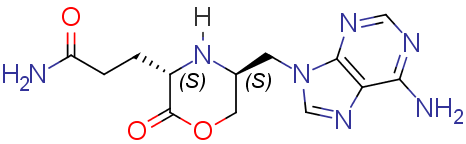 | 295.48 | 320.41 | 62.58 | 116.48 |
| 66 | 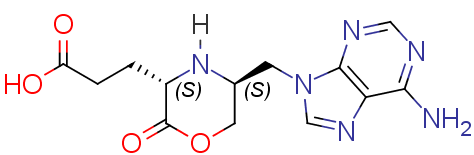 | 290.72 | 313.91 | 62.21 | 108.08 |
| 67 | 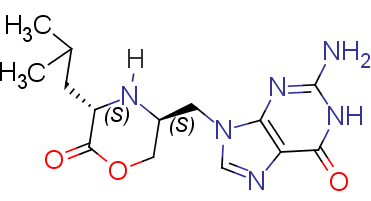 | 304.76 | 323.09 | 63.48 | 96.97 |
| 68 | 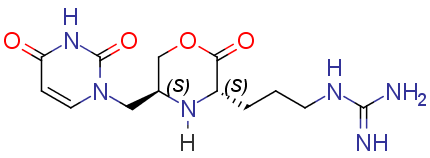 | 301.05 | 319.79 | 62.88 | 111.21 |
| 69 | 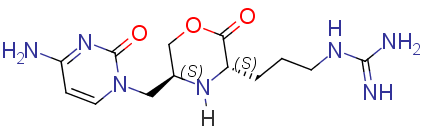 | 306.89 | 336.43 | 63.54 | 131.45 |
| 70 | 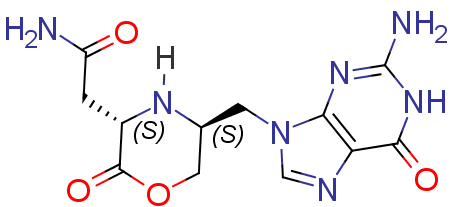 | 280.91 | 297.95 | 61.50 | 130.43 |
| 71 | 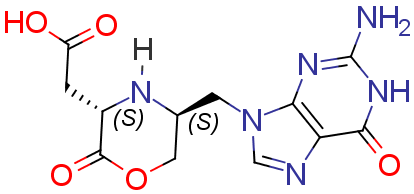 | 276.36 | 291.80 | 61.23 | 120.12 |
| 72 | 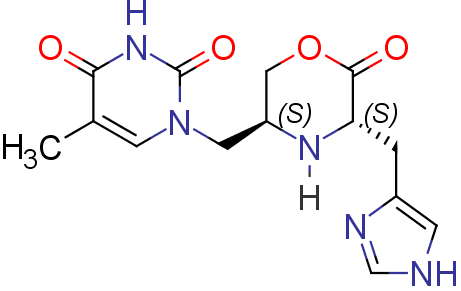 | 295.47 | 309.82 | 62.52 | 79.38 |
| 73 | 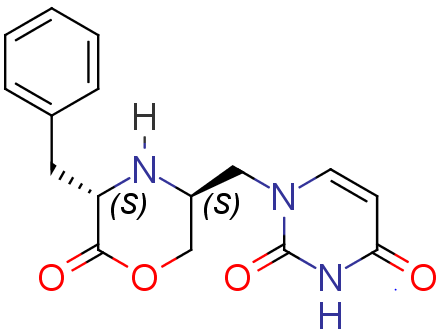 | 302.84 | 309.19 | 63.11 | 66.99 |
| 74 | 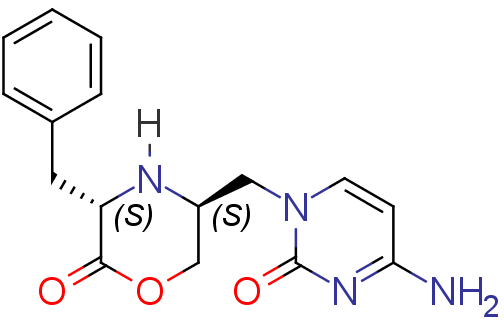 | 311.37 | 329.93 | 63.96 | 78.29 |
| 75 | 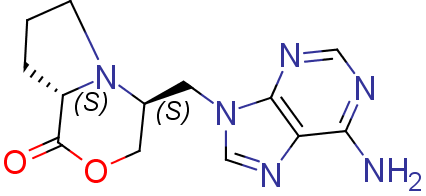 | 271.87 | 291.37 | 60.68 | 72.94 |
| 76 | 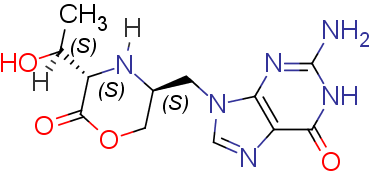 | 274.02 | 282.87 | 60.98 | 102.32 |
| 77 | 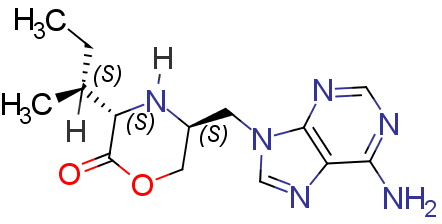 | 301.16 | 326.81 | 63.05 | 81.22 |
| 78 | 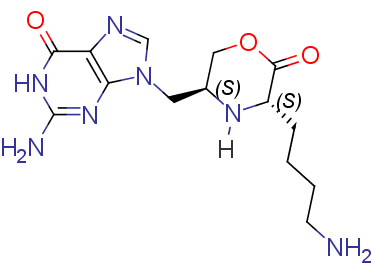 | 314.82 | 332.29 | 64.24 | 115.68 |
| 79 | 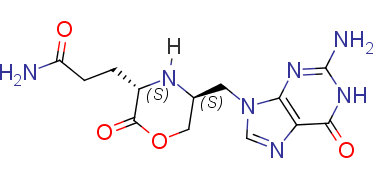 | 299.99 | 321.41 | 63.04 | 131.97 |
| 80 | 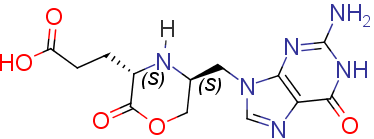 | 294.41 | 306.79 | 62.69 | 119.89 |
| 81 | 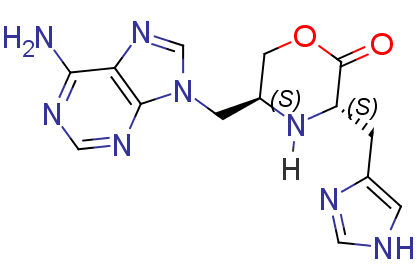 | 304.46 | 326.50 | 63.35 | 98.65 |
| 82 | 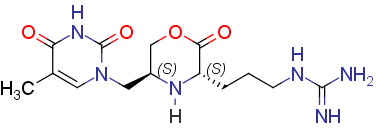 | 319.20 | 339.15 | 64.45 | 110.50 |
| 83 | 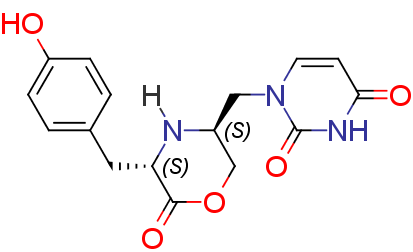 | 310.27 | 318.17 | 63.65 | 82.77 |
| 84 | 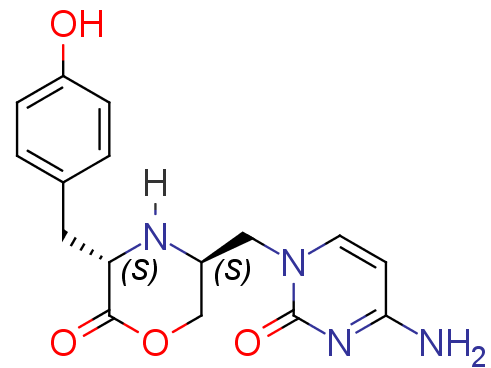 | 314.69 | 328.10 | 64.20 | 95.34 |
| 85 | 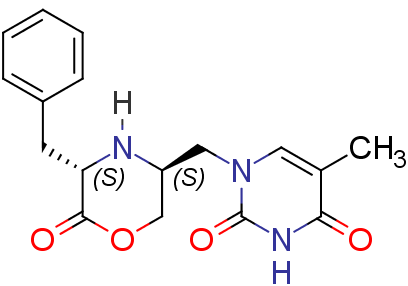 | 322.28 | 329.55 | 64.72 | 64.81 |
| 86 | 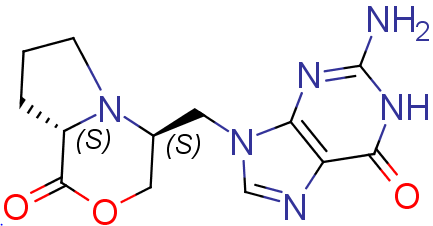 | 278.63 | 300.06 | 61.21 | 91.14 |
| 87 | 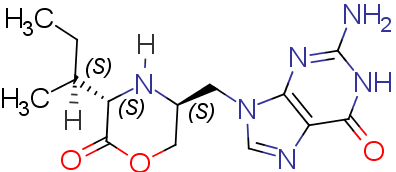 | 304.64 | 320.75 | 63.47 | 95.74 |
| 88 | 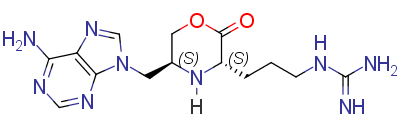 | 328.02 | 352.39 | 65.27 | 129.15 |
| 89 | 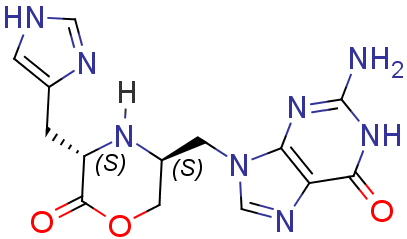 | 309.27 | 324.51 | 63.80 | 113.33 |
| 90 | 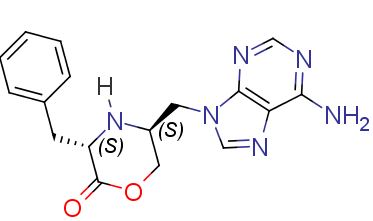 | 330.23 | 351.85 | 65.42 | 81.35 |
| 91 | 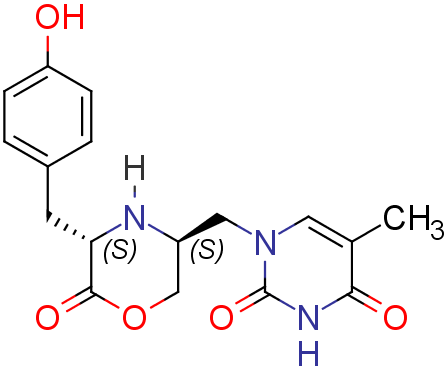 | 328.44 | 337.67 | 65.22 | 82.28 |
| 92 | 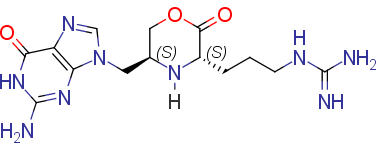 | 330.69 | 349.31 | 65.53 | 138.52 |
| 93 | 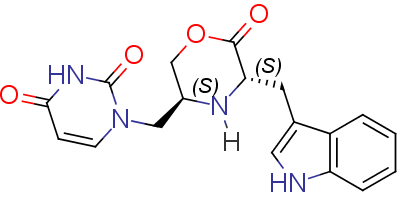 | 336.18 | 337.39 | 66.08 | 77.13 |
| 94 | 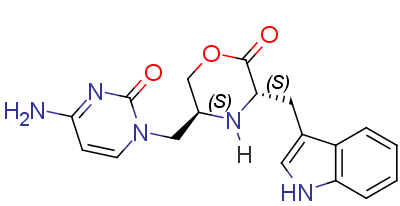 | 341.63 | 361.99 | 66.55 | 66.51 |
| 95 | 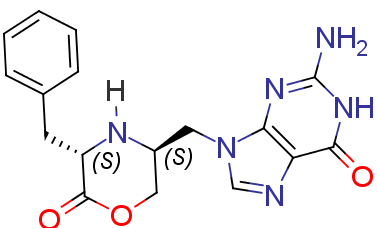 | 335.24 | 348.24 | 65.95 | 97.11 |
| 96 | 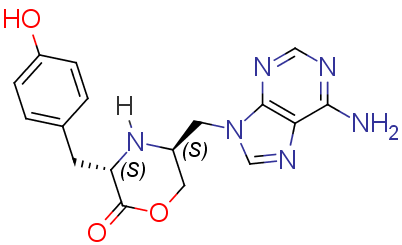 | 336.86 | 348.74 | 66.01 | 99.11 |
| 97 | 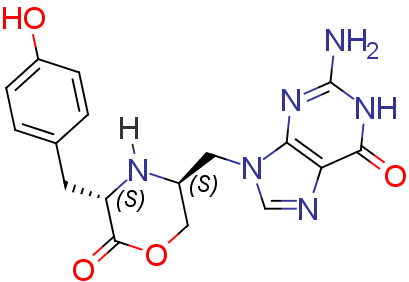 | 341.08 | 350.88 | 66.50 | 115.98 |
| 98 | 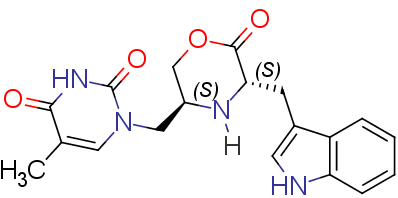 | 354.60 | 359.43 | 67.56 | 75.42 |
| 99 | 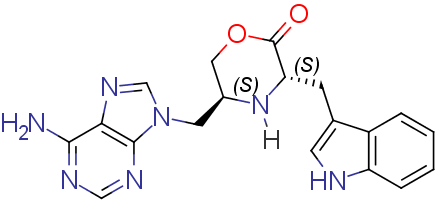 | 362.48 | 376.59 | 68.33 | 89.95 |
| 100 | 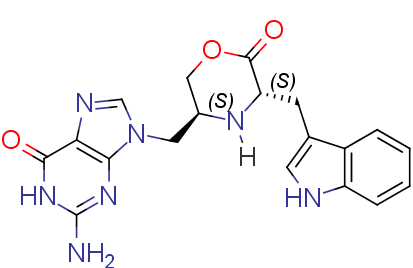 | 367.20 | 373.13 | 68.67 | 107.48 |
|  |  |  |  |  |  |

*Polar Surface Area

**S3. Pre-Tox analyses**

| Table S2. Toxicity Model Report for ABR | | | | | |
| --- | --- | --- | --- | --- | --- |
| **Entry** | **Classification** | **Target** | **Shorthand** | **Prediction** | **Probability** |
| 1 | Organ toxicity | Hepatotoxicity | dili | Inactive | 0.64 |
| 2 | Organ toxicity | Neurotoxicity | neuro | Active | 0.64 |
| 3 | Organ toxicity | Nephrotoxicity | nephro | Inactive | 0.64 |
| 4 | Organ toxicity | Respiratory toxicity | respi | Active | 0.87 |
| 5 | Organ toxicity | Cardiotoxicity | cardio | Inactive | 0.76 |
| 6 | Toxicity end points | Carcinogenicity | carcino | Inactive | 0.50 |
| 7 | Toxicity end points | Immunotoxicity | immuno | Active | 0.84 |
| 8 | Toxicity end points | Mutagenicity | mutagen | Inactive | 0.56 |
| 9 | Toxicity end points | Cytotoxicity | cyto | Inactive | 0.51 |
| 10 | Toxicity end points | BBB-barrier | bbb | Active | 0.79 |
| 11 | Toxicity end points | Ecotoxicity | eco | Inactive | 0.71 |
| 12 | Toxicity end points | Clinical toxicity | clinical | Active | 0.57 |
| 13 | Toxicity end points | Nutritional toxicity | nutri | Inactive | 0.56 |
| 14 | Tox21-Nuclear receptor signalling pathways | Aryl hydrocarbon Receptor (AhR) | nr_ahr | Inactive | 0.79 |
| 15 | Tox21-Nuclear receptor signalling pathways | Androgen Receptor (AR) | nr_ar | Inactive | 0.95 |
| 16 | Tox21-Nuclear receptor signalling pathways | Androgen Receptor Ligand Binding Domain (AR-LBD) | nr_ar_lbd | Inactive | 0.97 |
| 17 | Tox21-Nuclear receptor signalling pathways | Aromatase | nr_aromatase | Inactive | 0.89 |
| 18 | Tox21-Nuclear receptor signalling pathways | Estrogen Receptor Alpha (ER) | nr_er | Inactive | 0.93 |
| 19 | Tox21-Nuclear receptor signalling pathways | Estrogen Receptor Ligand Binding Domain (ER-LBD) | nr_er_lbd | Inactive | 0.98 |
| 20 | Tox21-Nuclear receptor signalling pathways | Peroxisome Proliferator Activated Receptor Gamma (PPAR-Gamma) | nr_ppar_gamma | Inactive | 0.95 |
| 21 | Tox21-Stress response pathways | Nuclear factor (erythroid-derived 2)-like 2/antioxidant responsive element (nrf2/ARE) | sr_are | Inactive | 0.94 |
| 22 | Tox21-Stress response pathways | Heat shock factor response element (HSE) | sr_hse | Inactive | 0.94 |
| 23 | Tox21-Stress response pathways | Mitochondrial Membrane Potential (MMP) | sr_mmp | Inactive | 0.83 |
| 24 | Tox21-Stress response pathways | Phosphoprotein (Tumor Supressor) p53 | sr_p53 | Inactive | 0.88 |
| 25 | Tox21-Stress response pathways | ATPase family AAA domain-containing protein 5 (ATAD5) | sr_atad5 | Inactive | 0.96 |
| 26 | Molecular Initiating Events | Thyroid hormone receptor alpha (THRα) | mie_thr_alpha | Inactive | 0.95 |
| 27 | Molecular Initiating Events | Thyroid hormone receptor beta (THRβ) | mie_thr_beta | Inactive | 0.70 |
| 28 | Molecular Initiating Events | Transtyretrin (TTR) | mie_ttr | Inactive | 0.83 |
| 29 | Molecular Initiating Events | Ryanodine receptor (RYR) | mie_ryr | Inactive | 0.85 |
| 30 | Molecular Initiating Events | GABA receptor (GABAR) | mie_gabar | Inactive | 0.73 |
| 31 | Molecular Initiating Events | Glutamate N-methyl-D-aspartate receptor (NMDAR) | mie_nmdar | Inactive | 0.98 |
|  | Molecular Initiating Events | alpha-amino-3-hydroxy-5-methyl-4-isoxazolepropionate receptor (AMPAR) | mie_ampar | Inactive | 0.98 |
| 33 | Molecular Initiating Events | Kainate receptor (KAR) | mie_kar | Inactive | 0.99 |
| 34 | Molecular Initiating Events | Achetylcholinesterase (AChE) | mie_ache | Inactive | 0.64 |
| 35 | Molecular Initiating Events | Constitutive androstane receptor (CAR) | mie_car | Inactive | 0.99 |
| 36 | Molecular Initiating Events | Pregnane X receptor (PXR) | mie_pxr | Active | 0.69 |
| 37 | Molecular Initiating Events | NADH-quinone oxidoreductase (NADHOX) | mie_nadhox | Inactive | 0.89 |
| 38 | Molecular Initiating Events | Voltage gated sodium channel (VGSC) | mie_vgsc | Inactive | 0.67 |
| 39 | Molecular Initiating Events | Na+/I- symporter (NIS) | mie_nis | Inactive | 0.82 |
| 40 | Metabolism | Cytochrome CYP1A2 | CYP1A2 | Inactive | 0.60 |
| 41 | Metabolism | Cytochrome CYP2C19 | CYP2C19 | Inactive | 0.67 |
| 42 | Metabolism | Cytochrome CYP2C9 | CYP2C9 | Inactive | 0.64 |
| 43 | Metabolism | Cytochrome CYP2D6 | CYP2D6 | Inactive | 0.50 |
| 44 | Metabolism | Cytochrome CYP3A4 | CYP3A4 | Inactive | 0.70 |
| 45 | Metabolism | Cytochrome CYP2E1 | CYP2E1 | Inactive | 0.98 |
| Table S3. Toxicity Model Report for AMO | | | | | |
| **Entry** | **Classification** | **Target** | **Shorthand** | **Prediction** | **Probability** |
| 1 | Organ toxicity | Hepatotoxicity | dili | Inactive | 0.78 |
| 2 | Organ toxicity | Neurotoxicity | neuro | Active | 0.82 |
| 3 | Organ toxicity | Nephrotoxicity | nephro | Active | 0.55 |
| 4 | Organ toxicity | Respiratory toxicity | respi | Active | 0.90 |
| 5 | Organ toxicity | Cardiotoxicity | cardio | Inactive | 0.84 |
| 6 | Toxicity end points | Carcinogenicity | carcino | Inactive | 0.51 |
| 7 | Toxicity end points | Immunotoxicity | immuno | Inactive | 0.87 |
| 8 | Toxicity end points | Mutagenicity | mutagen | Inactive | 0.57 |
| 9 | Toxicity end points | Cytotoxicity | cyto | Inactive | 0.71 |
| 10 | Toxicity end points | BBB-barrier | bbb | Active | 0.57 |
| 11 | Toxicity end points | Ecotoxicity | eco | Inactive | 0.61 |
| 12 | Toxicity end points | Clinical toxicity | clinical | Active | 0.68 |
| 13 | Toxicity end points | Nutritional toxicity | nutri | Inactive | 0.59 |
| 14 | Tox21-Nuclear receptor signalling pathways | Aryl hydrocarbon Receptor (AhR) | nr_ahr | Inactive | 0.93 |
| 15 | Tox21-Nuclear receptor signalling pathways | Androgen Receptor (AR) | nr_ar | Inactive | 0.99 |
| 16 | Tox21-Nuclear receptor signalling pathways | Androgen Receptor Ligand Binding Domain (AR-LBD) | nr_ar_lbd | Inactive | 0.99 |
| 17 | Tox21-Nuclear receptor signalling pathways | Aromatase | nr_aromatase | Inactive | 0.98 |
| 18 | Tox21-Nuclear receptor signalling pathways | Estrogen Receptor Alpha (ER) | nr_er | Inactive | 0.81 |
| 19 | Tox21-Nuclear receptor signalling pathways | Estrogen Receptor Ligand Binding Domain (ER-LBD) | nr_er_lbd | Inactive | 0.97 |
| 20 | Tox21-Nuclear receptor signalling pathways | Peroxisome Proliferator Activated Receptor Gamma (PPAR-Gamma) | nr_ppar_gamma | Inactive | 0.96 |
| 21 | Tox21-Stress response pathways | Nuclear factor (erythroid-derived 2)-like 2/antioxidant responsive element (nrf2/ARE) | sr_are | Inactive | 0.97 |
| 22 | Tox21-Stress response pathways | Heat shock factor response element (HSE) | sr_hse | Inactive | 0.97 |
| 23 | Tox21-Stress response pathways | Mitochondrial Membrane Potential (MMP) | sr_mmp | Inactive | 0.93 |
| 24 | Tox21-Stress response pathways | Phosphoprotein (Tumor Supressor) p53 | sr_p53 | Inactive | 0.91 |
| 25 | Tox21-Stress response pathways | ATPase family AAA domain-containing protein 5 (ATAD5) | sr_atad5 | Inactive | 0.89 |
| 26 | Molecular Initiating Events | Thyroid hormone receptor alpha (THRα) | mie_thr_alpha | Inactive | 0.95 |
| 27 | Molecular Initiating Events | Thyroid hormone receptor beta (THRβ) | mie_thr_beta | Inactive | 0.88 |
| 28 | Molecular Initiating Events | Transtyretrin (TTR) | mie_ttr | Inactive | 0.92 |
| 29 | Molecular Initiating Events | Ryanodine receptor (RYR) | mie_ryr | Inactive | 0.87 |
| 30 | Molecular Initiating Events | GABA receptor (GABAR) | mie_gabar | Inactive | 0.65 |
| 31 | Molecular Initiating Events | Glutamate N-methyl-D-aspartate receptor (NMDAR) | mie_nmdar | Inactive | 0.74 |
|  | Molecular Initiating Events | alpha-amino-3-hydroxy-5-methyl-4-isoxazolepropionate receptor (AMPAR) | mie_ampar | Inactive | 0.96 |
| 33 | Molecular Initiating Events | Kainate receptor (KAR) | mie_kar | Inactive | 0.99 |
| 34 | Molecular Initiating Events | Achetylcholinesterase (AChE) | mie_ache | Inactive | 0.54 |
| 35 | Molecular Initiating Events | Constitutive androstane receptor (CAR) | mie_car | Inactive | 0.99 |
| 36 | Molecular Initiating Events | Pregnane X receptor (PXR) | mie_pxr | Inactive | 0.75 |
| 37 | Molecular Initiating Events | NADH-quinone oxidoreductase (NADHOX) | mie_nadhox | Inactive | 0.99 |
| 38 | Molecular Initiating Events | Voltage gated sodium channel (VGSC) | mie_vgsc | Active | 0.55 |
| 39 | Molecular Initiating Events | Na+/I- symporter (NIS) | mie_nis | Inactive | 0.82 |
| 40 | Metabolism | Cytochrome CYP1A2 | CYP1A2 | Inactive | 0.88 |
| 41 | Metabolism | Cytochrome CYP2C19 | CYP2C19 | Inactive | 0.85 |
| 42 | Metabolism | Cytochrome CYP2C9 | CYP2C9 | Inactive | 0.65 |
| 43 | Metabolism | Cytochrome CYP2D6 | CYP2D6 | Inactive | 0.63 |
| 44 | Metabolism | Cytochrome CYP3A4 | CYP3A4 | Inactive | 0.72 |
| 45 | Metabolism | Cytochrome CYP2E1 | CYP2E1 | Inactive | 0.99 |
